# Assessing Human Genetic Variations in Glucose Transporter SLC2A10 and Their Role in Altering Structural and Functional Properties

**DOI:** 10.3389/fgene.2018.00276

**Published:** 2018-07-25

**Authors:** Michael T. Zimmermann, Raul Urrutia, Margot A. Cousin, Gavin R. Oliver, Eric W. Klee

**Affiliations:** ^1^Department of Health Science Research, Division of Biomedical Statistics and Informatics, Mayo Clinic, Rochester, MN, United States; ^2^Bioinformatics Research and Development Laboratory, Genomics Sciences and Precision Medicine Center, Medical College of Wisconsin, Milwaukee, WI, United States; ^3^Laboratory of Epigenetics and Chromatin Dynamics, Department of Biochemistry and Molecular Biology, Epigenomics Translational Program, Center for Individualized Medicine, Mayo Clinic, Rochester, MN, United States; ^4^Center for Individualized Medicine, Mayo Clinic, Rochester, MN, United States

**Keywords:** genetics, molecular modeling, natural variation, variant of uncertain significance, ATS

## Abstract

**Purpose:** Demand is increasing for clinical genomic sequencing to provide diagnoses for patients presenting phenotypes indicative of genetic diseases, but for whom routine genetic testing failed to yield a diagnosis. DNA-based testing using high-throughput technologies often identifies variants with insufficient evidence to determine whether they are disease-causal or benign, leading to categorization as variants of uncertain significance (VUS).

**Methods:** We used molecular modeling and simulation to generate specific hypotheses for the molecular effects of variants in the human glucose transporter, GLUT10 (*SLC2A10*). Similar to many disease-relevant membrane proteins, no experimentally derived 3D structure exists. An atomic model was generated and used to evaluate multiple variants, including pathogenic, benign, and VUS.

**Results:** These analyses yielded detailed mechanistic data, not currently predictable from sequence, including altered protein stability, charge distribution of ligand binding surfaces, and shifts toward or away from transport-competent conformations. Consideration of the two major conformations of GLUT10 was important as variants have conformation-specific effects. We generated detailed molecular hypotheses for the functional impact of variants in GLUT10 and propose means to determine their pathogenicity.

**Conclusion:** The type of workflow we present here is valuable for increasing the throughput and resolution with which VUS effects can be assessed and interpreted.

## Introduction

The metabolism of simple sugars is a fundamental biologic process involving multiple protein transporters for cellular uptake. Genetic variants in the GLUT (*SLC2A*) family of sugar transport proteins have been associated with common conditions such as type-2 diabetes and coronary heart disease, and can be causal for rare diseases. Each GLUT protein has a characteristic tissue-and subcellular localization-specific expression pattern (Joost et al., 2002), contributing to the phenotypic diversity associated with genetic variants. Understanding the molecular mechanisms underlying disease mediated by the GLUT family is important as clinical sequencing is uncovering novel variants of uncertain significance (VUS). Disambiguating VUS that may contribute to rare diseases is critical for maximizing the diagnostic yield and benefit of genomic sequencing.

Rare loss-of-function variants in *SLC2A10* (encoding GLUT10) are causal for arterial tortuosity syndrome (ATS) (Dawson et al., 2001). ATS is a rare connective tissue disorder characterized by tortuosity of the aorta and middle-sized arteries, as well as focal and widespread stenoses. Affected patients have increased risk of arterial aneurysms and dissections and also have connective tissue-related phenotypes overlapping TGFβ-related disorders such as Loeys-Dietz syndrome. Recently, GLUT10 was shown to transport dehydroascorbic acid (DAA), with this transport significantly reduced in ATS patient fibroblasts (Nemeth et al., 2016). The mechanistic underpinning of ATS are not completely understood, but are hypothesized to be insufficient ascorbic acid in the endoplasmic reticulum as an enzymatic cofactor for generating collagen- and elastin-stabilizing hydroxylases, resulting in a destabilized extracellular matrix (Segade, 2010). There are no gold-standard diagnostic laboratory tests or validated biomarkers for ATS, and diagnosis relies on clinical phenotypic findings and the identification of biallelic pathogenic variation in *SLC2A10*. Hence, a diagnosis of ATS is often hindered by the identification of VUS. Novel methodologies to assist VUS interpretation are needed.

Molecular dynamics (MD) leverages the atomic structure in physics-based and time-dependent simulations and have extensive use for interpreting the relationships between protein sequence, structure, dynamics, and function. van der Kamp et al. (2010), Sneha and Doss (2016), and Zimmermann et al. (2017) have applied these methods to characterize missense VUS. While GLUT10 has no experimentally solved structure, experimental structures for other GLUT-family proteins are available. We leveraged information from these structures to generate models of GLUT10 in two conformations that bookend the ligand transport process. Using these models, 23 missense variants in GLUT10 were characterized, predicting their effect on structural and dynamic properties of the protein. We analyzed each variant independently to give a detailed picture of how genetic variation may affect the GLUT10 protein function. This information may aid in variant interpretation and also generated hypotheses that can be functionally tested to subsequently clarify VUS function.

## Materials and Methods

### Editorial Policies and Ethical Considerations

This study is retrospective and analyzes the effect of genomic variants on an encoded protein. This study does not use patient data and conforms to institutional review boards and ethical guidelines.

### Multiple Sequence Alignment (MSA)

Paralogs of SLC2A1 and SLC2A10 were identified from the Ensembl database and the corresponding canonical transcript sequences downloaded from UniProt (Magrane and Consortium, 2011). Whole-family paralog and selected multi-species members of GLUT3, 5, and 10 were aligned using Clustal-Omega (Sievers et al., 2011) at the European Bioinformatics Institute with default settings. GLUT10 is conserved among mammals with 78% identity to mouse and rat orthologs. It exhibits low sequence identity among human paralogs with 24% identity to GLUT1 (*SLC2A1*), 27% identity to GLUT3 (*SLC2A3*) and 28% identity to GLUT5 (*SLC2A5*). Paralog sequences differ mostly for loops between transmembrane (TM) helices (Augustin, 2010). Conservation at each residue was calculated by Clustal-Omega and mapped to structural models using ConSurf (Ashkenazy et al., 2010). Protein sequence identifiers and experimental conformations, identified by manual literature review of related proteins (Deng et al., 2015), are listed in Supplementary Table S1. The MSA is available in Supplementary Table S2.

### Model Generation and Evaluation

Using templates from GLUT5, homology models of GLUT10 in two states along the transport pathway were generated using Modeller version 9.15. We named these conformations assuming GLUT10 localized to the plasma membrane; the channel is either inward-facing (IF) or outward-facing (OF). When GLUT10 is within the mitochondrial or ER membrane, the intracellular side would be cytoplasmic and the extracellular side within the organelle. Because experimentally derived structures are not available for the human proteins, the OF experimentally derived model of rat GLUT5 (4YBQ, Nomura et al., 2015; 3.27 Å resolution), and the IF model of bovine GLUT5 (4YB9, Nomura et al., 2015; 3.20 Å resolution) were used in homology modeling using the residue equivalences from the GLUT family MSA. We compared multiple experimental structures (resolution range, 1.5–3.8 Å) to our model to assess quality. We quantified structural similarity using C^α^ RMSD of TM helices calculated by CE (Shindyalov and Bourne, 2001).

Models were validated using standard metrics from multiple online servers including VADAR (Willard et al., 2003), MolProbity (Chen et al., 2010), and DisEMBL (Linding et al., 2003). Disorder was defined by all three DisEMBL metrics and confirmed by cross-references with regions unresolved in crystallographic studies. Our structural model was evaluated by the TM-specific software, QMEANBrane (Studer et al., 2014). We used InterProScan (Mitchell et al., 2015) version 5.15-54.0 for domain and linear motif prediction. The MESSA meta-server (Cong and Grishin, 2012) provided six different TM helix predictions; consensus TM prediction required prediction by at least three predictors. An α-D-glucose structure was downloaded from the ZINC database (Irwin et al., 2012) (ZINC03861213) and docked to each GLUT10 model using CDOCKER (Erickson et al., 2004) as implemented in Discovery Studio (BIOVIA, 2017).

### Modeled Variants

*SLC2A10* polymorphic variants (population MAF ≥ 0.01) were identified in ExAC (Dataset, 2015). Additional variants were selected from ClinVar (Landrum et al., 2014) and HGMD (Stenson et al., 2012). We grouped benign and likely benign variants together and similarly for pathogenic and likely pathogenic variants. Variants with conflicting annotations were considered VUS.

### Molecular Simulation

Molecular dynamics simulations were performed using the CHARMm c36b2 all-atom force-field (Cornell et al., 1995) with a 2fs time step. GLUT10’s long disordered loop was constrained using harmonic restraints to limit its motion. A simplified distance-dependent implicit environment in Discovery Studio (BIOVIA, 2017) was used with a dielectric constant of 80 and a pH of 7.4. Models were energy minimized for 2,000 steps using steepest decent followed by 4,000 steps of conjugate gradient and the SHAKE procedure. Each duplicate system was independently heated to 300 K over 300 ps and equilibrated for 2 ns followed by 25 ns production simulation (50 ns total, for each variant). Prior to analysis, each simulation was superposed onto the C^α^ atoms of TM helices from the initial WT conformation using by CE (Shindyalov and Bourne, 2001). Simulation data may be available upon request.

### Statistical Analysis

We measured the distances among sites of modeled variants and used these measures to group variants into spatially defined clusters. Spatial clustering was quantified by compactness, defined as the geometric mean of cluster sizes, and density, defined as the number of edges with clusters. We assessed significance using permutation. Clusters were visualized using a 2D network where amino acids were connected if they were within 15 Å. We tested if the spatial relationships among modeled variants differed from randomly distributed variants using permutation.

Distributions were compared to one another using *t*-tests. All time-dependent metrics were subsampled to 100 observations and the median *t*-statistic over 10 rounds of resampling used. We calculated principal component (PC) analysis of MD trajectories in Cartesian space using C^α^ atoms of the TM helices and their short connecting loops. For visualization, we used Kruskal’s non-metric multidimensional scaling (MDS) as implemented in the MASS package of R (Venables and Ripley, 2002).

The region of PC space capturing the densest 75% of data from simulations of benign variants was used to define regions commonly sampled. To compare variants along two PCs at once, we used a best-fit line and residual-based cutoff that captured the densest 60% of data from simulations of benign variants. In both cases, a variant was designated as “altered” in the corresponding PC if its median value was outside of the region defined by benign variants.

Because of the distinct differences in the ends of helices H4 and H10 between the OF and IF conformations, we chose residues at the ends of these helices as conformational monitors. The distance between residue pairs S124 and S436 or L101 and L413 monitor the extent of channel opening at the intracellular or extracellular sides, respectively. These four residues also form six angles that indicate the relative orientation of these two helices. Correlations among structure- and dynamics-based metric were computed using Spearman correlation and were adjusted using the Benjamini-Hochberg procedure.

### Software

We downloaded annotations for sequence-based predictive algorithms, such as SIFT (Kumar et al., 2009) and CADD (Kircher et al., 2014), from dbNSFP (Liu et al., 2015). We used Discovery Studio (Eswar et al., 2006; BIOVIA, 2017) v2017 to generate initial models. We used the bio3d (Grant et al., 2006) R package version 2.2.4 for analysis. Molecular visualizations were generated in PyMol (Cannone et al., 2017) version 1.8.7 and VMD (Humphrey et al., 1996) version 1.9.3. Changes in folding energy upon mutation were computed using FoldX (Van Durme et al., 2011) version 4. The protein coding effect of DNA variants was translated using CAVA (Münz et al., 2015) version 1.2.3.

## Results

### Structural Model and Validation

An atomic model including 91% of GLUT10 amino acids (residues 10 through 499) was generated and analyzed with multiple algorithms for comprehensive quality evaluation. The remaining residues form a cytoplasmic domain that lacks sufficient experimental data for modeling. For our model, all bond length, angle, hydrogen bond geometries, electrostatic and VdW scores are within typical ranges; 94% of residues were within the core Ramachandran regions and 98% within allowed regions (Supplementary Figure S1). Quality comparison to experimental transmembrane proteins identified unfavorable dihedral angles and residue packing metrics within the disordered loop (residues 315 through 395). This was expected and the loop was restrained in simulations in order to limit its contribution to the differences seen between variants. Consensus sequence-based TM predictions agree with our model (Supplementary Figure S2). Charged and hydrophilic residues were positioned outside of the TM regions, with exceptions internal to the ligand-transport channel. Thus, we believe our GLUT10 model is of high quality.

### Comparison to Other Experimental Protein Structures

Our GLUT10 models and experimental structures of other GLUT family proteins were compared to one another in order to assess model consistency (Supplementary Table S3). After energy minimization, our IF model remained close to its bovine GLUT5 template (4YB9; 1.51 Å RMSD) and the IF structure of human GLUT1 (4PYP; 1.98 Å RMSD). The two IF experimental structures were also similar to each another (1.26 Å RMSD). Our OF model differed moderately from the rat GLUT5 template (2.70 Å RMSD) and the OF structure of human GLUT3 (4ZWC; 2.96 Å RMSD). The two OF experimental structures differed less from each other (1.94 Å RMSD). In all cases, IF models had closer agreement with IF structures, than with OF structures, and vice versa. Comparing across additional GLUT family experimental structures, our models had smaller deviation from one another (2.59 Å RMSD) than the deviations between experimental IF and OF structures (3.01–3.95 Å RMSD). Selected superimposed structures are shown in Supplementary Figure S3. Thus, the deviations of our models from the experimental structures used as templates are conservative and of a similar magnitude as observed among other experimental structures.

### Selection of Variants

To facilitate the comparison and interpretation of modeling results for variants of uncertain significance, benign and pathogenic variants were selected and modeled. The benign variant set included three variants reported as benign in ClinVar, including A106S (minor allele frequency, MAF = 0.003), A206T (MAF = 0.101), and A385G (MAF = 0.013). Additionally, we included L475F which is reported by ExAC (MAF = 0.012) in the BQSRTrancheSNP99.90to99.95 and is not included in a low-complexity or homopolymer repeat region. Ten variants were identified as pathogenic and included R132W, S81R, G142V, R231Q, R231H, G246E, G426W, E437K, G445E, and R105C. Nine VUSs were also selected for characterization, including: E48K, L85P, R132Q, F168L, R225H, R231W, Q243K, S476F, and G489R.

### Structural Organization of GLUT10 and Variants

**FIGURE 1:**
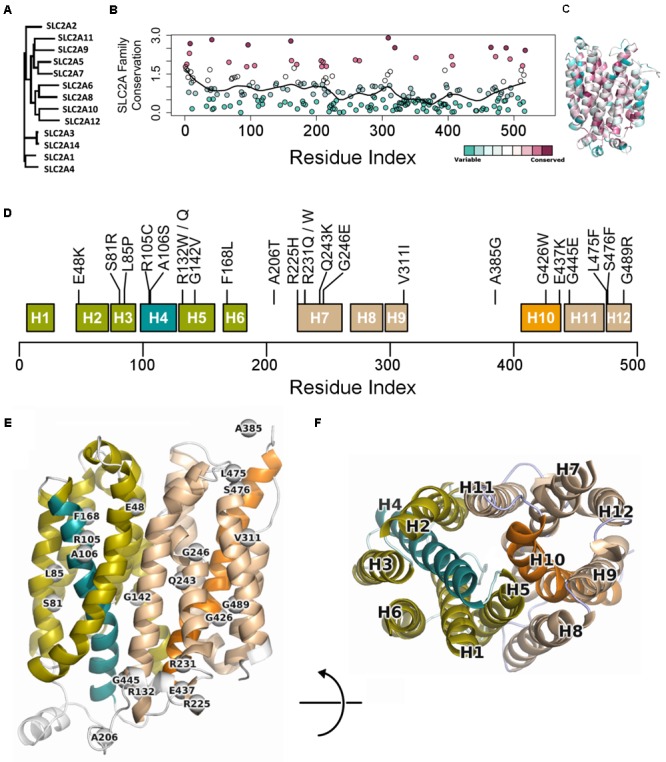
Conservation across human paralogs of GLUT10. **(A)** Phylogenetic tree indicating the relative amino acid sequence relatedness of paralogs to each other. **(B)** The per-residue conservation scores using the SLC2A family MSA and as computed by ConSurf, are shown along with a smoothed average (black line). Regions of high conservation are distributed across the sequence. **(C)** Our model of IF SLC2A10 is shown colored by ConSurf conservation. While more highly conserved regions are distributed across the sequence, they tend to coincide with the inner portions of the channel that will interact with ligands. **(D)** Locations of the 23 GLUT10 variants studied. Some amino acids have multiple observed alterations at the same site, such as R132W and R132Q. GLUT10 is organized into two sub-domains or helical bundles, colored olive and tan. Each bundle is organized around H4 or H10, highlighted in teal and orange, respectively. **(E)** The modeled variants are distributed throughout the GLUT10 structure as depicted on our IF model, but tend to occur on the intracellular side and within the core of each helical bundle. **(F)** TM helices are numbered according to their sequential order and viewed from the intracellular side.

To better understand the role of each residue in GLUT10 function, we considered sequence conservation among human paralogs in sequence and 3D (**Figures 1A–C**). Sites of conservation are distributed throughout the sequence, but pack between TM-helices, or make up ligand-binding sites of the channel lining. The 12-helix architecture is organized into two bundles of six helices – the 4th and 10th alpha helices (H4 and H10, respectively) each pass through one of these bundles (**Figure 1D**). The ends of H4 and H10 make up part of the “gates” that oppositely open on one end and close on the other.

Variants are distributed across the linear sequence, but fall into three regions of the structure (**Figures 1E,F** and Supplementary Figure S4). We considered the 3D relationships between the variants and generated a 2D network to aid interpretation of their relative proximity. Three groups of variants are discernable from this analysis, including one group inside each of the two helical bundles and a third at the IF opening. These clusters were compact (see the section “Materials and Methods”; *p* = 0.091; Supplementary Figure S5). Clusters were significantly dense after normalizing by compactness (*p* = 0.023).

We extracted each helical bundle, calculated electrostatic surfaces and revealed strong charge segregation between these surfaces (Supplementary Figure S6). Variants within these regions may cause region-specific likelihoods of altering protein stability, allosteric communication, ligand affinity, or selectivity. The channel lining is largely defined by the interaction between these two surfaces, indicating the likely important role of charge segregation along the channel lining.

### Structural Features Affected by Missense Variants

**FIGURE 2:**
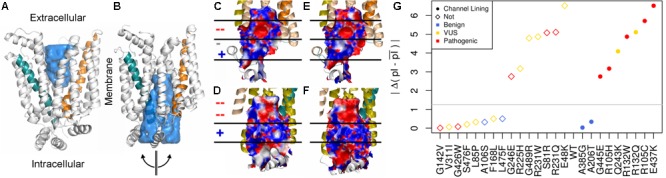
Conservation of inner pore properties. Despite the relatively modest sequence identity across the GLUT family, structural properties, such as the electrostatic distribution around and within the ligand-binding pocket, are conserved. We show our **(A)** outward-facing and **(B)** inward-facing GLUT10 models with the binding pocket filled in. In the outward-facing conformation, a large cavity is accessible from the extracellular side, but the inner binding site is fully occluded. A semi-transparent blue surface fills the solvent accessible cavity. Helix 4 (teal) and helix 10 (orange) are colored similar to previous figures. After transitioning to the inward-facing conformation, an opposite relationship is observed. Considering the inward-facing conformation, we split the structure in half according to the two helical bundled described above and color each interior surface by electrostatic potential (red negative, white neutral, and blue positive). Pealing the structure in half reveals that the interior of the pore for **(C,D)** GLUT10 and **(E,F)** GLUT1 are highly similar, despite their low sequence conservation (24%) and that GLUT1 was not used in the MSA for constructing our GLUT10 model (Supplementary Table S2). Horizontal lines and positive (+) or negative (–) annotation indicate regions where electrostatics switch consistently between GLUT1 and GLUT10. **(G)** Pathogenic variants tend to alter charge segregation of the channel lining. We first selected residue positions that make up the channel interior lining in either conformation. Each variant’s change to the electrostatic character of the channel interior was quantified using a simple pI-based score; see the section “Materials and Methods” section. A gray line marks the maximum change possible among uncharged amino acids.

The surface properties of the channel lining are largely conserved across the GLUT family. We visualized conservation of channel lining’s electrostatic surface by comparing our model of GLUT10 to an available experimental structure of GLUT1. GLUT1 sequences were not included in developing the GLUT10 model. The segregation of the electrostatic surface is visually apparent (**Figure 2**) and consistent between these proteins despite low sequence identity (24%). The primary ligand-binding pocket is the most consistent feature of the channel lining. It is a broad negatively charged region, deep within the channel, and accessible to the exterior in both conformations. We studied the effect of pathogenic variants on the channel lining’s electrostatic surface. Half of the tested pathogenic variants in the channel lining affect the electrostatic surface, while none of the tested benign variants within the channel lining do (**Figure 2G**). Additionally, VUSs in the channel lining (R132Q and Q243K) affect the electrostatic surface in a manner more consistent with observations of the pathogenic variants than of benign variants.

**FIGURE 3:**
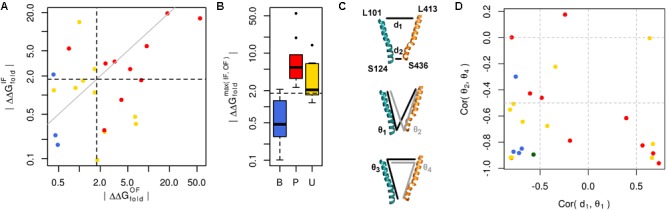
Many pathogenic variants destabilize the protein structure. **(A)** Variants of each class are clearly separated from one another by their effect on protein folding energy. We calculated ΔΔG_fold_ for each variant using our structural models of both the IF and OF conformations. Black dashed lines indicate ΔΔG = 3 k_B_T, while the gray line indicates equivalence. L475F was omitted from the scatterplot because it had a near-zero ΔΔG_fold_ in both conformations. **(B)** Significant differences are observed between each group of variants with known pathogenic variants exhibiting greater ΔΔG_fold_ than benign variants (*p* = 0.031). Many VUSs also destabilize the protein structure (*p* = 0.016). Known pathogenic variants were not significantly more destabilizing than VUSs (*p* = 0.910). **(C)** Variants often disrupt communication through the structure. Residues at the ends of H4 and H10, colored as in previous figures, are used as conformational monitors. **(D)** For the IF WT model as a representative example and benign variants, there is a strong negative correlation between the angle, 𝜃_1_, and distance, d_1_. This relationship indicates that as 𝜃_1_ increases, corresponding to opening of the intracellular side, d_1_ decreases, corresponding to the closure of the extracellular side. Similar relationships are observed for the opposite pattern, such as the negative correlation between the angles 𝜃_2_ and 𝜃_4_.

We used our GLUT10 structural models to predict each variant’s impact on protein stability, as measured by ΔΔG_fold_. Because destabilization of either conformation could alter protein function, we considered each conformation as well as the maximum ΔΔG_fold_ across conformations. Energy distributions illustrated a significant separation between pathogenic and benign variants (**Figure 3A** and Supplementary Figures S7, S8). This separation was dependent on the protein conformation studied. Many of the modeled VUSs destabilize GLUT10 similar to pathogenic variants. Unlike pathogenic variants, a subset of modeled VUSs showed low destabilization, more closely resembling benign variants (**Figure 3B**). Therefore, computational assessment of variant- and conformation-specific ΔΔG_fold_ differentiated pathogenic from benign variants and could be used to rank VUS.

**FIGURE 4:**
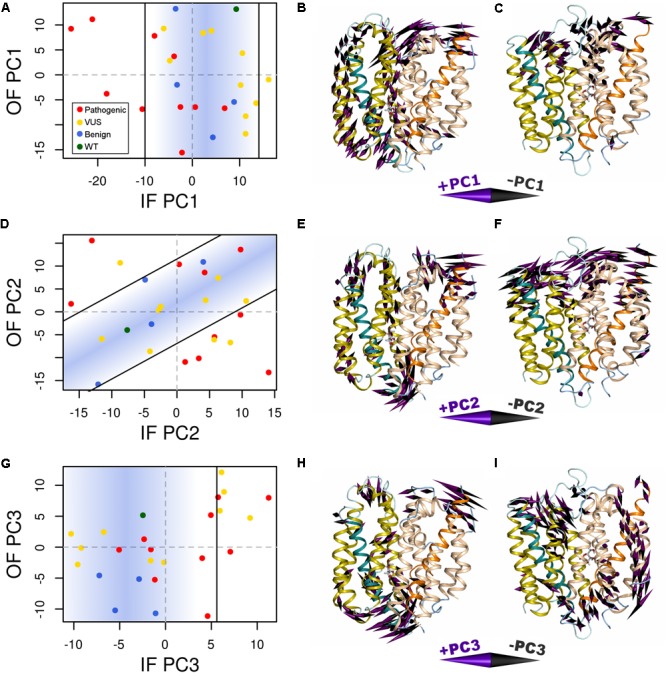
Variants alter conformational dynamics. The conformational dynamics within our MD trajectories of GLUT10 were evaluated using PCA. **(A)** The median PC1 coordinate of each variant from each initial conformation is shown. Specific variants shifted the inward-facing (IF) dynamics in the negative direction further than was observed in any of the simulations of benign variants or the WT, while there is no clear separation between types of variants from the outward-facing (OF) dynamics. Black lines indicate the bounds of the densest 75% of WT and benign simulations, and a blue gradient is used to highlight this same region; see the section “Materials and Methods.” Variants whose median values are outside of this area are considered altered in the PC motion. **(B)** We visualized the IF PC1 in 3D by placing a cone at each residue that is proportional to its magnitude and direction. Small motions are omitted for clarity. The positive direction of IF PC1 indicates motion of the intracellular sides of H1, H3, and H4 away from the C-terminal bundle with shifting of H5 upward toward the extracellular side and motion of the extracellular sides of H8 and H10 away from the N-terminal bundle. These motions coincide with some of the transitions toward the OF conformation. **(C)** We visualized the OF PC1 in the same way. The dominant feature of this motion is movement of the extracellular sides of H5, H7, and H8. These motions coincide with some of the transitions toward the IF conformation. The motions apparent in **(D–F)** PC2 and **(G–I)** PC3 of each conformation are visualized in the same way. For each PC, we identified a line of discrimination that separated regions of PC space occupied by WT and benign variants from regions dominated by pathogenic variants.

Variant-specific effects on conformational dynamics were tested using MD simulation. Each variant was simulated in duplicate in both the IF and OF conformations. GLUT10 structure and dynamics were summarized using PCs (**Figure 4**; see the section “Materials and Methods”). The first PCs tend to describe collective motions which may be related to the conformational change between OF and IF. Pathogenic variants could function by changing how collective GLUT10 is when transitioning between conformations. To track coordination through the structure, a set of reference distances and angles along TM helices 4 and 7 were measured during our simulations (**Figures 3C,D**). For the WT protein, these measures were negatively correlated between IF and OF conformations – the IF must move toward the OF and vice versa – an observation that is in agreement with *a priori* expectation. Certain variants were associated with substantial changes in the correlations among these angles – an allosteric effect modulated by the variant. Five variants diminished the magnitude of correlation [three pathogenic and two VUS with Cor(d_1_,𝜃_1_) between -0.5 and 0.0]. Seven others altered the sign of correlation [four pathogenic and three VUS with Cor(d_1_,𝜃_1_) > 0.0], decoupling the structural features of the IF-to-OF transition. Simulations indicate that these variants may alter how GLUT10 moves in association with ligand binding.

**FIGURE 5:**
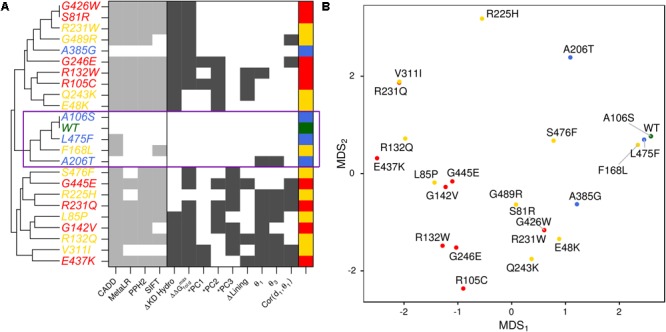
Structure-based metrics discriminate pathogenic from benign variants and provide mechanistic hypothesis of altered function. **(A)** The most informative structure-based metrics presented in previous figures were discretized and compared to commonly used genomics-based predictive algorithms (see the section “Materials and Methods”). Metrics from both perspectives effectively discriminate between pathogenic and benign variants. However, structure-based metrics provide mechanistic hypotheses for how the protein is altered by each variant. Additionally, they indicate more variable effects for VUSs than genomics-based predictors. **(B)** We visualized the similarity of variants to one another using MDS.

We summarized the structure- and dynamics-based alterations induced by 23 variants modeled in this study using MDS and clustering (**Figure 5** and Supplementary Figure S9). WT and the benign variants A106S and L475F are not associated with alteration of any of the structure-based metrics we have considered. They are consistently clustered together and with the VUS F168L, which was also not associated with changes in these metrics. The benign variant A385G is associated with altered hydrophobicity and stability, leading to its clustering with other pathogenic variants and VUSs that alter these properties. However, it is more similar to WT and other benign variants than any pathogenic variants are. Pathogenic variants tended to cluster together. These structure-based annotations provide detailed hypothesis-generating information for the interpretation of variants.

## Discussion

While the functional importance of GLUT10 (*SLC2A10*) and its causal link in the rare monogenic disorder, ATS, are well established, additional resources to aid VUS interpretation for GLUT10 are needed. There is currently no experimentally derived structural model for GLUT10 from which additional studies could be designed. Here, we describe the first protein modeling study of GLUT10. Sufficient structural data on other GLUT family proteins existed to enable us to generate GLUT10 protein model predictions for two conformations. We applied detailed computational evaluation of genetic variants to better understand the underlying mechanisms of known pathogenic variants and which VUSs may affect the protein in similar ways. Using these models in unbiased simulations, we assessed alterations in protein structure and dynamics. The primary aims of this study were to evaluate the ability to inform pathogenicity of VUSs using protein model-based predictions in the context of known pathogenic and benign variants and to generate hypotheses of functional effects that could be tested in subsequent lab-based studies.

Atomic structural modeling enables *in silico* evaluation of variant impact at a resolution difficult to obtain using experimental methods, particularly for membrane proteins. Molecular modeling and simulation methods may provide insights for cases where genomic testing results are negative or inconclusive. However, these methods are not commonly applied in translational settings for multiple reasons. High-resolution experimental models are not always available. Constructing *in silico* atomic models is time consuming and not always amenable to clinical timelines. The multiple degrees of freedom in molecular simulations present a challenge of false discovery – the concern that observed difference are coincidence of sampling, the identified changes are not mechanistically relevant, or the findings may not be robust and reproducible. Likewise, there are likely many biologically real ways of altering a protein that are not sufficient to result in disease. Distinguishing disease-relevant alterations from among the many potential alterations to protein dynamics is a major research challenge. The alterations identified by simulation are challenging to validate in biological systems, to determine how specific or sensitive computations tests are for determining pathogenicity. Finally, the necessary expertise is not often found in the translational clinical settings to carry out these modeling studies. Continued evaluation of variants and refinements of such models may allow for these techniques to contribute more directly to the evidence supporting or refuting variant pathogenicity, but currently *in silico* protein modeling is not included in the variant interpretation guidelines as functional evidence (Richards et al., 2015). We believe that molecular modeling holds tremendous value for the clinical interpretation of missense variants identified from high-throughput sequencing studies. As we show in this study, specific hypotheses can be generated for the molecular effects of variants. This is valuable for better understanding the underlying mechanisms of known pathogenic variants, but also for the interpretation of VUS.

We addressed several of the above challenges in VUS interpretation by focusing on summary metrics such as geometric distances and PCs. We believe this approach is more robust and reproducible compared to analyses that focus exclusively on alteration of specific interactions as there are likely multiple ways to alter protein dynamics. The molecular modeling of variants in GLUT10 gave valuable information for planning how to address the physiologic effects of each. For example, our analysis of the VUS E48K showed that it specifically shifted the protein conformation away from WT as quantified by PC2. In our model, E48 is on the OF side of the protein and nearby in space to D468. The charge repulsion from this pair in the WT may balance stability between the IF and OF conformations. E48K appears to stabilize one conformation, likely leading to loss of function. The pathogenic variant E437K is positioned at the IF of the protein and E437 interacts closely in space with three arginine residues: R130, R132, and R296. E437K likely disrupts these interactions, leading to our prediction of alteration in PC1. R132 is also the site of pathogenic variants, both of which alter PC2. Similar to E48K, E437K shifts the protein conformation to favor one side of the transport process. However, E437K was also predicted to destabilize the protein structure, alter allosteric communication, and is a charge-alteration within the channel lining. Therefore, there is greater evidence supporting E437K as a loss-of-function variant, compared to E48K. However, a specific hypothesis with similar features at the molecular level is shared between the pathogenic E437K and VUS E48K. This underscores the fact there are molecular alterations that affect the protein shared between pathogenic variants and VUS. When the overlap is partial, it is challenging to distinguish those that are disease-causal from among them. Establishing a specific and testable mechanistic hypothesis by which a VUS may affect the protein similarly to a pathogenic variant, as we have provided an example for here, demonstrates the value of molecular modeling for variant interpretation. Thus, molecular modeling provides a rationale for prioritizing experiments to confirm whether or not E48K is a loss-of-function variant, and also mechanisms for interpreting those experiments.

The models we have made in this work are a first step toward a molecular and mechanistic understanding of genetic variation in GLUT10. Our predictions could be validated using experimental tests. Altered folding could be assessed using protein abundance in cellular membranes by isolating the membranes and running targeted mass spectroscopy. A simpler, but still challenging approach would be to quantify changes in circular dichroism spectra, which has advanced in recent years for membrane proteins (Miles and Wallace, 2016). Our predicted alterations in PCs could be assayed by directed labeling techniques and nuclear magnetic resonance spectroscopy (McDermott, 2009). Alternatively, they could be assessed by introducing cysteine mutations at carefully selected sites and differential crosslinking as measured by mass spectroscopy. Our predictions of altered channel lining as well as the overall function of GLUT10 could be assessed by ability to bind and transport glucose or DAA. Membranes containing GLUT10 could be synthesized and used to make vesicles that could pump glucose of DAA inside (Tian et al., 2017) with differential uptake indicative of altered protein function. While functional follow-up is beyond the scope of the current study, we have demonstrated the potential for molecular modeling of GLUT10 variant and of variants identified by high-throughput sequencing in general, to inform clinical interpretation and prioritization of functional tests.

Additional techniques are available for testing specific hypotheses *in silico*; initiating molecular modeling from hypothesis-driven research is likely to generate clearer findings within a pre-determined scope. For example, to specifically test how variants affect the ease with which GLUT10 transitions between OF and IF, a series of targeted simulations could be made that guide the simulation between the two conformations and differences in the transition paths or energies analyzed. To more directly test the hypothesis that ligand passage through the channel is affected by a variant, a series of steered simulations could be made that guide ligand through the channel. Therefore, the scope and resolution of modeling studies are broad and can be tailored to address specific hypotheses.

Changes to the native structures like those investigated here are one of the many modes by which a variant could alter protein function. For example, a variant could affect interactions with other molecules without destabilizing the native structure. These additional modes of alteration emphasize the need for more sensitive and comprehensive tools for the functional and context-specific interpretation of genetic variants. Currently, molecular modeling is not necessarily ready to be a clinical diagnostic tool – it depends on the resolution of available data and the extent of relevant physiologic details captured by the model. In many cases, modeling is a means of reducing the unsolved case rate by directing further testing downstream of genomic results through a more detailed molecular understanding of the effects of genomic alterations. We believe that molecular modeling will become an increasingly important consideration when attempting to interpret the clinical relevance of novel variants.

## Conclusion

We have demonstrated the potential for molecular modeling to bring improved understanding of the functional consequences of genomic variants discovered using high-throughput sequencing, in the membrane protein GLUT10. We have highlighted some of the critical considerations in the translation of molecular modeling and simulation to the interpretation of clinically observed VUSs. These include the necessity of considering multiple functional conformations of the protein as a variant may affect each differently. We believe the integration of this type of information into the interpretation of VUS will become a critical aspect of Precision Medicine.

## Author Contributions

MZ designed the study, carried out analyses, generated figures, and wrote the paper. RU designed the study, wrote the paper, and contributed to review. MC and EK contributed to clinical assessment, writing, and review. GO contributed to writing and review. EK contributed to clinical assessment, writing, and review.

## Conflict of Interest Statement

The authors declare that the research was conducted in the absence of any commercial or financial relationships that could be construed as a potential conflict of interest.

## References

[B1] Ashkenazy H. Erez E. Martz E. Pupko T. Ben-Tal N. (2010). ConSurf 2010: calculating evolutionary conservation in sequence and structure of proteins and nucleic acids. *Nucleic Acids Res.* 38 W529–W533. 10.1093/nar/gkq399 20478830 PMC2896094

[B2] Augustin R. (2010). The protein family of glucose transport facilitators: it’s not only about glucose after all. *IUBMB Life* 62 315–333. 10.1002/iub.315 20209635

[B3] BIOVIA (2017). *Dassault Systèmes BIOVIA, Discovery Studio Modeling Environment, Release 2017.* San Diego: Dassault Systèmes.

[B4] Cannone G. Visentin S. Palud A. Henneke G. Spagnolo L. (2017). *The PyMOL Molecular Graphics System. Version 1.5.0.3.* New York, NY: Schrödinger, LLC.

[B5] Chen V. B. Arendall W. B. III Headd J. J. Keedy D. A. Immormino R. M. Kapral G. J. (2010). MolProbity: all-atom structure validation for macromolecular crystallography. *Acta Crystallogr. D Biol. Crystallogr.* 66(Pt 1) 12–21. 10.1107/S0907444909042073 20057044 PMC2803126

[B6] Cong Q. Grishin N. V. (2012). MESSA: MEta-Server for protein sequence analysis. *BMC Biol.* 10:82. 10.1186/1741-7007-10-82 23031578 PMC3519821

[B7] Cornell W. D. Bayly C. I. Gould I. R. Merz K. M. Ferguson D. M. Spellmeyer D. C. (1995). A second generation force field for the simulation of proteins, nucleic acids, and organic molecules. *J. Am. Chem. Soc.* 117 5179–5197. 10.1021/ja00124a002

[B8] Dataset (2015). *Exome Aggregation Consortium (ExAC).* Available at: http://exac.broadinstitute.org

[B9] Dawson P. A. Mychaleckyj J. C. Fossey S. C. Mihic S. J. Craddock A. L. Bowden D. W. (2001). Sequence and functional analysis of GLUT10: a glucose transporter in the type 2 diabetes-linked region of chromosome 20q12-13.1. *Mol. Genet. Metab.* 74 186–199. 10.1006/mgme.2001.3212 11592815

[B10] Deng D. Sun P. Yan C. Ke M. Jiang X. Xiong L. (2015). Molecular basis of ligand recognition and transport by glucose transporters. *Nature* 526 391–396. 10.1038/nature14655 26176916

[B11] Erickson J. A. Jalaie M. Robertson D. H. Lewis R. A. Vieth M. (2004). Lessons in molecular recognition: the effects of ligand and protein flexibility on molecular docking accuracy. *J. Med. Chem.* 47 45–55. 10.1021/jm030209y 14695819

[B12] Eswar N. Webb B. Marti-Renom M. A. Madhusudhan M. S. Eramian D. Shen M. Y. (2006). Comparative protein structure modeling using modeller. *Curr. Protoc. Bioinformatics* 15 5.6.1–5.6.30. 10.1002/0471250953.bi0506s15 18428767 PMC4186674

[B13] Grant B. J. Rodrigues A. P. ElSawy K. M. McCammon J. A. Caves L. S. (2006). Bio3d: an R package for the comparative analysis of protein structures. *Bioinformatics* 22 2695–2696. 10.1093/bioinformatics/btl461 16940322

[B14] Humphrey W. Dalke A. Schulten K. (1996). VMD: visual molecular dynamics. *J. Mol. Graph.* 14 33–38. Epub 1996/02/01 10.1016/0263-7855(96)00018-5 8744570

[B15] Irwin J. J. Sterling T. Mysinger M. M. Bolstad E. S. Coleman R. G. (2012). ZINC: a free tool to discover chemistry for biology. *J. Chem. Inf. Model.* 52 1757–1768. 10.1021/ci3001277 22587354 PMC3402020

[B16] Joost H. G. Bell G. I. Best J. D. Birnbaum M. J. Charron M. J. Chen Y. T. (2002). Nomenclature of the GLUT/SLC2A family of sugar/polyol transport facilitators. *Am. J. Physiol. Endocrinol. Metab.* 282 E974–E976. 10.1152/ajpendo.00407.2001 11882521

[B17] Kircher M. Witten D. M. Jain P. O’Roak B. J. Cooper G. M. Shendure J. (2014). A general framework for estimating the relative pathogenicity of human genetic variants. *Nat. Genet.* 46 310–315. 10.1038/ng.2892 24487276 PMC3992975

[B18] Kumar P. Henikoff S. Ng P. C. (2009). Predicting the effects of coding non-synonymous variants on protein function using the SIFT algorithm. *Nat. Protoc.* 4 1073–1081. 10.1038/nprot.2009.86 19561590

[B19] Landrum M. J. Lee J. M. Riley G. R. Jang W. Rubinstein W. S. Church D. M. (2014). ClinVar: public archive of relationships among sequence variation and human phenotype. *Nucleic Acids Res.* 42 D980–D985. 10.1093/nar/gkt1113 24234437 PMC3965032

[B20] Linding R. Jensen L. J. Diella F. Bork P. Gibson T. J. Russell R. B. (2003). Protein disorder prediction: implications for structural proteomics. *Structure* 11 1453–1459. 10.1016/j.str.2003.10.002 14604535

[B21] Liu X. Wu C. Li C. Boerwinkle E. (2015). dbNSFP v3.0: a one-stop database of functional predictions and annotations for human non-synonymous and splice site SNVs. *Hum. Mutat.* 37 235–241. 10.1002/humu.22932 26555599 PMC4752381

[B22] Magrane M. Consortium U. (2011). UniProt knowledgebase: a hub of integrated protein data. *Database* 2011:bar009. 10.1093/database/bar009 21447597 PMC3070428

[B23] McDermott A. (2009). Structure and dynamics of membrane proteins by magic angle spinning solid-state NMR. *Annu. Rev. Biophys.* 38 385–403. 10.1146/annurev.biophys.050708.133719 19245337

[B24] Miles A. J. Wallace B. A. (2016). Circular dichroism spectroscopy of membrane proteins. *Chem. Soc. Rev.* 45 4859–4872. 10.1039/c5cs00084j 27347568

[B25] Mitchell A. Chang H. Y. Daugherty L. Fraser M. Hunter S. Lopez R. (2015). The interpro protein families database: the classification resource after 15 years. *Nucleic Acids Res.* 43 D213–D221. 10.1093/nar/gku1243 25428371 PMC4383996

[B26] Münz M. Ruark E. Renwick A. Ramsay E. Clarke M. Mahamdallie S. (2015). CSN and CAVA: variant annotation tools for rapid, robust next-generation sequencing analysis in the clinic. CSHL preprint server. *Genome Med.* 7:76 10.1101/016808 PMC4551696 26315209

[B27] Nemeth C. E. Marcolongo P. Gamberucci A. Fulceri R. Benedetti A. Zoppi N. (2016). Glucose transporter type 10-lacking in arterial tortuosity syndrome-facilitates dehydroascorbic acid transport. *FEBS Lett.* 590 1630–1640. 10.1002/1873-3468.12204 27153185

[B28] Nomura N. Verdon G. Kang H. J. Shimamura T. Nomura Y. Sonoda Y. (2015). Structure and mechanism of the mammalian fructose transporter GLUT5. *Nature* 526 397–401. 10.1038/nature14909 26416735 PMC4618315

[B29] Richards S. Aziz N. Bale S. Bick D. Das S. Gastier-Foster J. (2015). Standards and guidelines for the interpretation of sequence variants: a joint consensus recommendation of the American College of Medical Genetics and Genomics and the Association for Molecular Pathology. *Genet. Med.* 17 405–424. 10.1038/gim.2015.30 25741868 PMC4544753

[B30] Segade F. (2010). Glucose transporter 10 and arterial tortuosity syndrome: the vitamin C connection. *FEBS Lett.* 584 2990–2994. 10.1016/j.febslet.2010.06.011 20547159

[B31] Shindyalov I. N. Bourne P. E. (2001). A database and tools for 3-D protein structure comparison and alignment using the combinatorial extension (CE) algorithm. *Nucleic Acids Res.* 29 228–229. 10.1093/nar/29.1.228 11125099 PMC29823

[B32] Sievers F. Wilm A. Dineen D. Gibson T. J. Karplus K. Li W. (2011). Fast, scalable generation of high-quality protein multiple sequence alignments using clustal omega. *Mol. Syst. Biol.* 7:539. 10.1038/msb.2011.75 21988835 PMC3261699

[B33] Sneha P. Doss C. G. (2016). Molecular dynamics: new frontier in personalized medicine. *Adv. Protein Chem. Struct. Biol.* 102 181–224. 10.1016/bs.apcsb.2015.09.004 26827606

[B34] Stenson P. D. Ball E. V. Mort M. Phillips A. D. Shaw K. Cooper D. N. (2012). The human gene mutation database (HGMD) and its exploitation in the fields of personalized genomics and molecular evolution. *Curr. Protoc. Bioinformatics* 39 1.13.1–1.13.20. 10.1002/0471250953.bi0113s39 22948725

[B35] Studer G. Biasini M. Schwede T. (2014). Assessing the local structural quality of transmembrane protein models using statistical potentials (QMEANBrane). *Bioinformatics* 30 i505–i511. 10.1093/bioinformatics/btu457 25161240 PMC4147910

[B36] Tian X. Ye M. Cao Y. Wang C. (2017). Losartan improves palmitate-induced insulin resistance in 3T3-L1 adipocytes through upregulation of src phosphorylation. *Exp. Clin. Endocrinol. Diabetes* 125 136–140. 10.1055/s-0042-120709 28008588

[B37] van der Kamp M. W. Schaeffer R. D. Jonsson A. L. Scouras A. D. Simms A. M. Toofanny R. D. (2010). Dynameomics: a comprehensive database of protein dynamics. *Structure* 18 423–435. 10.1016/j.str.2010.01.012 20399180 PMC2892689

[B38] Van Durme J. Delgado J. Stricher F. Serrano L. Schymkowitz J. Rousseau F. (2011). A graphical interface for the foldx forcefield. *Bioinformatics* 27 1711–1712. 10.1093/bioinformatics/btr254 21505037

[B39] Venables W. N. Ripley B. D. (2002). *Modern Applied Statistics with S.* 4th Edn. New York, NY: Springer 10.1007/978-0-387-21706-2

[B40] Willard L. Ranjan A. Zhang H. Monzavi H. Boyko R. F. Sykes B. D. (2003). VADAR: a web server for quantitative evaluation of protein structure quality. *Nucleic Acids Res.* 31 3316–3319. 10.1093/nar/gkg565 12824316 PMC168972

[B41] Zimmermann M. T. Urrutia R. Oliver G. R. Blackburn P. R. Cousin M. A. Bozeck N. J. (2017). Molecular modeling and molecular dynamic simulation of the effects of variants in the TGFBR2 kinase domain as a paradigm for interpretation of variants obtained by next generation sequencing. *PLoS One* 12:e0170822. 10.1371/journal.pone.0170822 28182693 PMC5300139

