# Supplementary material for: Assessing Human Genetic Variations in Glucose Transporter SLC2A10 and Their Role in Altering Structural and Functional Properties
Source: Front Genet. 2018 Jul 25;9:276. doi: 10.3389/fgene.2018.00276 (PMC6068234; doi:10.3389/fgene.2018.00276)
Supplement: Supplementary file 1 [file Data_Sheet_1.DOCX]

Supplemental Information

Assessing Human Genetic Variations in Glucose Transporter SLC2A10 and Their Role in Altering Structural and Functional Properties

**Authors**: Michael T. Zimmermann^1,2^, Raul Urrutia^2,3,*^, Margot A. Cousin^4^, Gavin R. Oliver^1,4^, Eric W. Klee^1,4,*^

**Affiliations:**

^1^Department of Health Science Research, Division of Biomedical Statistics and Informatics, Mayo Clinic, Rochester, MN, USA

^2^Genomics Sciences and Precision Medicine Center, Medical College of Wisconsin, Milwaukee, WI, USA

^3^Laboratory of Epigenetics and Chromatin Dynamics, Department of Biochemistry and Molecular Biology, Epigenomics Translational Program, Center for Individualized Medicine, Mayo Clinic, Rochester, MN, USA

^4^Center for Individualized Medicine, Mayo Clinic, Rochester, MN, USA

^*^Corresponding author electronic address: rurrutia@mcw.edu, klee.eric@mayo.edu

**Table S1: Sequence and structure IDs for selected GLUT family proteins utilized in this study.**

| **Species** | **HGNC Symbol** | **UniProt ID** | **UniProt Accession** | **3D Structure^†^** | **Conformation** | **Ligand** |
| --- | --- | --- | --- | --- | --- | --- |
| Human | SLC2A1 | P11166 | GTR1_HUMAN | 4PYP (3.17)(1) | Inward | BNG |
| Human | SLC2A3 | P11169 | GTR3_HUMAN | **4ZW9** (1.50)(2)  4ZWC (2.60)(2) | **Out-Occl** Outward | Glucose Maltose |
| Human | SLC2A5 | P22732 | GTR5_HUMAN | - | - | - |
| Human | SLC2A10 | O95528 | GTR10_HUMAN | - | - | - |
| Mouse | slc2a3 | P32037 | GTR3_MOUSE | - | - | - |
| Mouse | slc2a5 | Q9WV38 | GTR5_MOUSE | - | - | - |
| Mouse | slc2a10 | Q8VHD6 | GTR10_MOUSE | - | - | - |
| Rat | slc2a3 | Q07647 | GTR3_RAT | - | - | - |
| Rat | slc2a5 | P43427 | GTR5_RAT | **4YBQ** (3.27)(3) | **Outward** |  |
| Rat | slc2a10 | D3ZVY8 | D3ZVY8_RAT | - | - | - |
| Bovine | slc2a5 | P58353 | GTR5_BOVINE | **4YB9** (3.20)(3) | **Inward** | apo |
| e.coli | XYLE | P0AGF4 | XYLE_ECOLI | 4JA3 (3.80)(4)  4GBY (2.81)(5) | Inward-Occl  Out-Occl | Xylose  Xylose |
| e.coli | FucP | J7R4Y2 | J7R4Y2_ECOLX | 3O7Q (3.14)(6) | Outward | Fructose |

^†^ PDB identifiers. A hyphen indicates no experimental structure is available at the time of writing. Crystallographic resolution (in Å) indicated in parenthesis followed by journal reference.

Structure IDs for SLC2A3 and SLC2A5 that were used as templates for our SLC2A10 modeling are colored similar to the resulting models presented in Figure S3 that indicate the conformational changes between each major conformation.

**Table S2: MSA for GLUT3, 5, and 10 from human (h), rat (r), and mouse (m).** This MSA was used for the residue equivalences between proteins for molecular modeling. The first row shows the secondary structure (SS; alpha-Helix, Loop, or intrinsically Disordered) from our model of hSLC2A10 in the outward-open conformation. Sites with variants analyzed in this study are indicated above secondary structure. For regions with insertions or deletions and low overall conservation, there may be multiple ways to place gaps with comparable score.

hSLC2A10 SNV K R P

hSLC2A10 SS -------LL—LLLHHHHHHHHHHH-HHHHHHHHHHHHHLLLLLLLLLLLLLLLL--------------LHHHHHHHHHHHHHHHHHHHHHHHHHHHLHHHHHHHHHHHHHHHHHHHHH--

hSLC2A10 -------MG--HSPPVLPLCASVS-LLGGLTFGYELAVISGALLPLQLDFGLSC--------------LEQEFLVGSLLLGALLASLVGGFLIDCYGRKQAILGSNLVLLAGSLTLGL--

rSlc2a10 -------MG--LGPTVLPLCASVS-LLGGLTFGYELAVISGALLPLQLDFGLSC--------------LEQELLVGSLLLGAFLASLVGGFLIDCYGRRRAILGSNVVLLAGSLILGL--

mSlc2a10 -------MG--LRPAVLLLCASVS-LLGGLTFGYELAVISGALLPLQLNFGLSC--------------LEQELLVGSLLLGALLASLVGGFLIDCYGRRRAILGSNAVLLAGSLILGL--

hSLC2A3 -------MGTQKVTPALIFAITVA-TIGSFQFGYNTGVINAPEKIIKEFINKTLTDKGNAPPSEVLLTSLWSLSVAIFSVGGMIGSFSVGLFVNRFGRRNSMLIVNLLAVTGGCFMGLCK

mSlc2a3 -------MGTTKVTPSLVFAVTVA-TIGSFQFGYNTGVINAPETILKDFLNYTLEERLEDLPSEGLLTALWSLCVAIFSVGGMIGSFSVGLFVNRFGRRNSMLLVNLLAIIAGCLMGFAK

rSlc2a3 -------MGTAKVTPSLVFAVTVA-TIGSFQFGYNTGVINAPETIIKDFLNYTLEERLEDLPSEGLLTTLWSLCVAIFSVGGMIGSFSVGLFVNRFGRRNSMLLVNLIAILGGCLMGFAK

hSLC2A5 MEQQDQSMKEGRLTLVLALATLIAAFGSSFQYGYNVAAVNSPALLMQQFYNETYYGRTGEFMEDFPLTLLWSVTVSMFPFGGFIGSLLVGPLVNKFGRKGALLFNNIFSIVPAILMGCSR

mSlc2a5 ME-EKHQEETGELTLVLALATLIAAFGSSFQYGYNVAAVNSPSEFMQQFYNDTYYDRNEENIESFTLTLLWSLTVSMFPFGGFIGSLMVGTLVNKLGRKGALLFNNIFSILPAILMGCSQ

rSlc2a5 ME-KEDQEKTGKLTLVLALATFLAAFGSSFQYGYNVAAVNSPSEFMQQFYNDTYYDRNKENIESFTLTLLWSLTVSMFPFGGFIGSLMVGFLVNNLGRKGALLFNNIFSILPAILMGCSK

* :. :: ..: :**: ..:.. :: : .. *. : .*.::.*: * ::: **: ::* * . : . :*

hSLC2A10 SNV Q

hSLC2A10 SNV CS W V L

hSLC2A10 SS -LLLHHHHHHHHHHHHHHHHHHHHHHHHHHHHHLLHHHHHHHHHHHHHHHHHHHHHL--LHHHHHHHLLLLLLHHHHHHHHHHHHHHHHHHHHLLLLL--------LLHHHHHHHHHHHL

hSLC2A10 -AGSLAWLVLGRAVVGFAISLSSMACCIYVSELVGPRQRGVLVSLYEAGITVGILLS--YALNYALAGTPWGWRHMFGWATAPAVLQSLSLLFLPAGT--------DETATHKDLIPLQG

rSlc2a10 -AGSLPWLLLGRSSVGFAISLSSMACCIYVSELVGPRQRGVLVSLYEVGITVGILFS--YGLNYVLAGRPWGWRHMFGWAAAPALLQSLSLFFLPAGA--------EGTAARQDLIPLQG

mSlc2a10 -ASSLPWLLLGRLSVGFAISLSSMACCIYVSELVGPRQRGVLVSLYEVGITVGILFS--YGLNYVLAGSPWGWRHMFGWAAAPALLQSLSLFLLPAGA--------EGTAAPKDLIPLQG

hSLC2A3 VAKSVEMLILGRLVIGLFCGLCTGFVPMYIGEISPTALRGAFGTLNQLGIVVGILVAQIFGLEFIL-GSEELWPLLLGFTILPAILQSAALPFCPESPRFLLINRKEEENAKQILQRLWG

mSlc2a3 IAESVEMLILGRLLIGIFCGLCTGFVPMYIGEVSPTALRGAFGTLNQLGIVVGILVAQIFGLDFIL-GSEELWPGLLGLTIIPAILQSAALPFCPESPRFLLINKKEEDQATEILQRLWG

rSlc2a3 IAESVEMLILGRLIIGIFCGLCTGFVPMYIGEVSPTALRGAFGTLNQLGIVVGILVAQVFGLDFIL-GSEELWPGLLGLTIIPAILQSAALPFCPESPRFLLINRKEEDQATEILQRLWG

hSLC2A5 VATSFELIIISRLLVGICAGVSSNVVPMYLGELAPKNLRGALGVVPQLFITVGILVAQIFGLRNLL-ANVDGWPILLGLTGVPAALQLLLLPFFPESPRYLLIQKKDEAAAKKALQTLRG

mSlc2a5 IAQSFELIIISRLLVGICAGISSNVVPMYLGELAPKNLRGALGVVPQLFITVGILVAQLFGLRSLL-ANEDGWPVLLGLTGVPAGLQLLLLPFFPESPRYLLIQKKDEAAAERALQTLRG

rSlc2a5 IAKSFEIIIASRLLVGICAGISSNVVPMYLGELAPKNLRGALGVVPQLFITVGILVAQLFGLRSVL-ASEEGWPILLGLTGVPAGLQLLLLPFFPESPRYLLIQKKNESAAEKALQTLRG

* *. :: .* :*: .:.: :*:.*: **.: : : *.****.: :.* * . * ::* : ** ** * : * . : : . * * *

hSLC2A10 SNV Q

hSLC2A10 SNV T H W K E I

hSLC2A10 SS LLLLLL-----------LLLLLLLLHHHHHHLHHHHHHHHHHHHHHHHHHHHHLHHHHHHHHHHHHHHHLLLLLLHHHHHHHHHHHHHHHHHHHHHHHHHHHLHHHHHHHHHHHHHHHHH

hSLC2A10 GEAPKL-----------GPGRPRYSFLDLFRARDNMRGRTTVGLGLVLFQQLTGQPNVLCYASTIFSSVGFHGGSSAVLASVGLGAVKVAATLTAMGLVDRAGRRALLLAGCALMALSVS

rSlc2a10 GETSKL-----------GLVKAQYTFLDLFRAQDGMWSRTVVGLGLVLFQQLTGQPNVLYYASTIFRSVGFHGGSSAVLASVGLGTVKVVATLIATGLVDRVGRRALLLSGCALMALSVS

mSlc2a10 RETSKP-----------GLVKPQYSFLDLFRAQDGMWSRTVVGLGLVLFQQLTGQPNVLYYASTIFRSVGFHGGSSAVLASVGLGTVKVAATLVATGLVDRAGRRVLLLFGCALMALSVS

hSLC2A3 TQDVSQDIQEMKDESARMSQEKQVTVLELFRVSSYRQPI-IISIVLQLSQQLSGINAVFYYSTGIFKDAGVQE---PIYATIGAGVVNTIFTVVSLFLVERAGRRTLHMIGLGGMAFCST

mSlc2a3 TSDVVQEIQEMKDESVRMSQEKQVTVLELFRSPNYVQPL-LISIVLQLSQQLSGINAVFYYSTGIFKDAGVQE---PIYATIGAGVVNTIFTVVSLFLVERAGRRTLHMIGLGGMAVCSV

rSlc2a3 TPDVIQEIQEMKDESIRMSQEKQVTVLELFKSPSYFQPL-LISVVLQLSQQFSGINAVFYYSTGIFQDAGVQE---PIYATIGAGVVNTIFTVVSLFLVERAGRRTLHMIGLGGMAVCSV

hSLC2A5 WDSVDREVAEIRQEDEAEKAAGFISVLKLFRMRSLRWQL-LSIIVLMGGQQLSGVNAIYYYADQIYLSAGVPEEH-VQYVTAGTGAVNVVMTFCAVFVVELLGRRLLLLLGFSICLIACC

mSlc2a5 WKDVHLEMEEIRKEDEAEKAAGFISVWKLFTMQSLRWQL-ISMIVLMAGQQLSGVNAIYYYADQIYLSAGVKSDD-VQYVTAGTGAVNVFMTILTIFVVELWGRRFLLLVGFSTCLIACL

rSlc2a5 WKDVDMEMEEIRKEDEAEKAAGFISVWKLFRMQSLRWQL-ISTIVLMAGQQLSGVNAIYYYADQIYLSAGVKSND-VQYVTAGTGAVNVFMTMVTVFVVELWGRRNLLLIGFSTCLTACI

:. .** . : * **::* : *: *: ..*. .: * *.*:. *. : :*: *** * : * . .

hSLC2A10 SNV G W

hSLC2A10 SS HHDDDDDDDDDDDDDDDDDDDDDDDDDDDDDDDDDDDDDDDDDDDDDDDDDDDDDDDDDDDDDDDDDDDDDDDDDHHHHHDDDDDDDDDDLLLHHHHHHHHHHHHHHHHHHHHHHHHHHH

hSLC2A10 GIGLVSFAVPMDSGPSCLAVPNATGQTGLPGDSGLLQDSSLPPIPRTNEDQREPILSTAKKTKPHPRSGDPSAPPRLALSSALPGPPLPARGHALLRWTALLCLMVFVSAFSFGFGPVTW

rSlc2a10 GIGLVSFAVSLDSGPSCLATSNTSQQVDLPQTPGLLVRTSLPPVLHTSGDQGQPVRSVTERPI-HP-VITASLGPALNIDSSVPTSPI--PEHTLLCWSALVCMMVYVSAFSFGFGPVTW

mSlc2a10 GIGLVSFAVSLDSGPSCLATSNASQQVDLPGSSGLLVRSSLPPVLHTNGDQGQLVLSVTERPI-HP-VITASLGPVLNTASPVPTSPI--LEHTLLCWSALVCMMVYVSAFSVGFGPVTW

hSLC2A3 LMT-----------------------------------------------------------------------VSLLLK----------DNYNGMSFVCIGAILVFVAFFEIGPGPIPW

mSlc2a3 FMT-----------------------------------------------------------------------ISLLLK----------DDYEAMSFVCIVAILIYVAFFEIGPGPIPW

rSlc2a3 FMT-----------------------------------------------------------------------ISLLLK----------DEYEAMSFVCIVAILVYVAFFEIGPGPIPW

hSLC2A5 VLT-----------------------------------------------------------------------AALALQ----------DTVSWMPYISIVCVISYVIGHALGPSPIPA

mSlc2a5 VLT-----------------------------------------------------------------------AALALQ----------NTISWMPYISIVCVIVYVIGHALGPSPIPA

rSlc2a5 VLT-----------------------------------------------------------------------VALALQ----------NTISWMPYVSIVCVIVYVIGHAVGPSPIPA

: * : : .: .:: :* . .* .*:

hSLC2A10 SNV K E FF R

hSLC2A10 SS HHHHHHLLLLHHHHHHHHHHHHHHHHHHHHHHHHHHHHHHHLHHHHHHHHHHHHHHHHHHHHHLLLLLLLLHHHHHHHHHHHHHL---------------------------

hSLC2A10 LVLSEIYPVEIRGRAFAFCNSFNWAANLFISLSFLDLIGTIGLSWTFLLYGLTAVLGLGFIYLFVPETKGQSLAEIDQQFQKRRFTLSFGHRQNS---TGIPYSRIEISAAS

rSlc2a10 LVLSEIYPVEIRGRAFAFCSSFNWAANLFISLSFLDLIGAIGLAWTFLLYGLTAVLGLAFIYLLVPETKGQSLAEIEQQFQMRRFPLSFGHRQH----SGIQYRRLEVSSAS

mSlc2a10 LVLSEIYPAEIRGRAFAFCSSFNWAANLFISLSFLDLIGAIGLAWTFLLYGLTAVLGLAFIYLLVPETKGQSLAEIEQQFQTSRFPLNFGHRQR----IGIQYHRLDVSSAS

hSLC2A3 FIVAELFSQGPRPAAMAVAGCSNWTSNFLVGLLFPSAAHYLG-AYVFIIFTGFLITFLAFTFFKVPETRGRTFEDITRAFEGQAHGADRSGKDGVMEMNSIEPAKETTTNV-

mSlc2a3 FIVAELFSQGPRPAAIAVAGCCNWTSNFLVGMLFPSAAAYLG-AYVFIIFAAFLIFFLIFTFFKVPETKGRTFEDIARAFEGQAHSGKGPA---GVELNSMQPVKETPGNA-

rSlc2a3 FIVAELFSQGPRPAAMAVAGCSNWTSNFLVGMFFPSAAAYLG-AYVFIIFAAFLVFFLIFTFFKVPETKGRTFEDITRAFEGQAQSGKGSA---GVELNSMQPVKETPGNA-

hSLC2A5 LLITEIFLQSSRPSAFMVGGSVHWLSNFTVGLIFPFIQEGLG-PYSFIVFAVICLLTTIYIFLIVPETKAKTFIEINQIFTKMNKVSEVYPEKEE--LKELPPVTSEQ----

mSlc2a5 LLITEIFLQSSRPAAYMIGGSVHWLSNFTVGLIFPFIQMGLG-PYSFIIFATICFLTTIYIFMVVPETKGRTFIEINQIFTMKNKVSDVYPKKEE-ELGALPHAILEQ----

rSlc2a5 LFITEIFLQSSRPSAYMIGGSVHWLSNFIVGLIFPFIQVGLG-PYSFIIFAIICLLTTIYIFMVVPETKGRTFVEINQIFAKKNKVSDVYPEKEEKELNDLPPATREQ----

:.::*:: * * . . .* :*: :.: * :* : *::: . : :: ****:.::: :* : * . :

**Table S3**: RMSDs among GLUT family protein structures. We compared each experimental structure and our GLUT10 models to each other using C_α_ atoms of the TM helices and the CE algorithm. The matrix is symmetric with the lower region filled gray as a visual guide. Bold font is used for comparisons between each model and their respective template. Template names are colored as in Table S1.

|  |  | IF | IF | **IF** | OF | OF | **OF** |
| --- | --- | --- | --- | --- | --- | --- | --- |
|  |  | GLUT10 | 4PYP | **4YB9** | GLUT10 | 4ZWC | **4YBQ** |
| IF | GLUT10 | 0.00 | 1.98 | **1.51** | 2.59 | 3.33 | 3.17 |
| IF | 4PYP | 1.98 | 0.00 | 1.26 | 3.95 | 3.13 | 3.91 |
| **IF** | **4YB9** | **1.51** | 1.26 | 0.00 | 3.25 | 3.01 | 3.40 |
| OF | GLUT10 | 2.59 | 3.95 | 3.25 | 0.00 | 2.96 | **2.70** |
| OF | 4ZWC | 3.33 | 3.13 | 3.01 | 2.96 | 0.00 | 1.94 |
| **OF** | **4YBQ** | 3.17 | 3.91 | 3.40 | **2.70** | 1.94 | 0.00 |


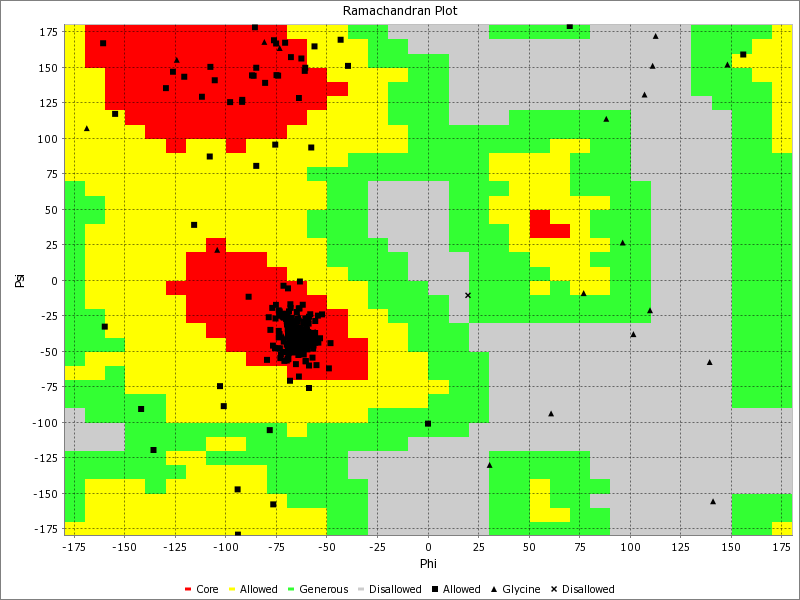

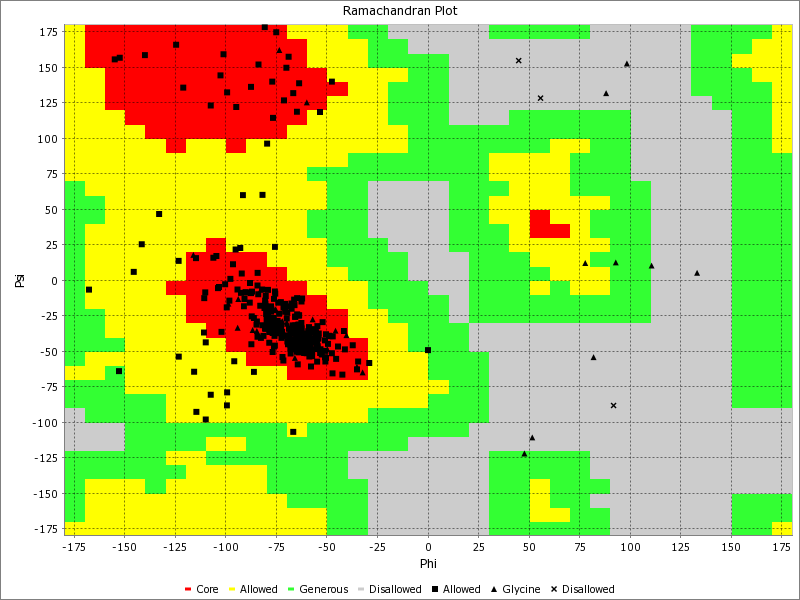


B

A

A

**Figure S1: Structural model evaluation metrics indicate that our initial model is of high quality.** A) Ramachandran plot (image copied from VADAR(7)at vadar.wishartlab.com) of the IF model indicates that 94% of residues are within the core regions and 98% within allowed regions. B) for the OF model, 92% of residues are within core regions and 98% within allowed.


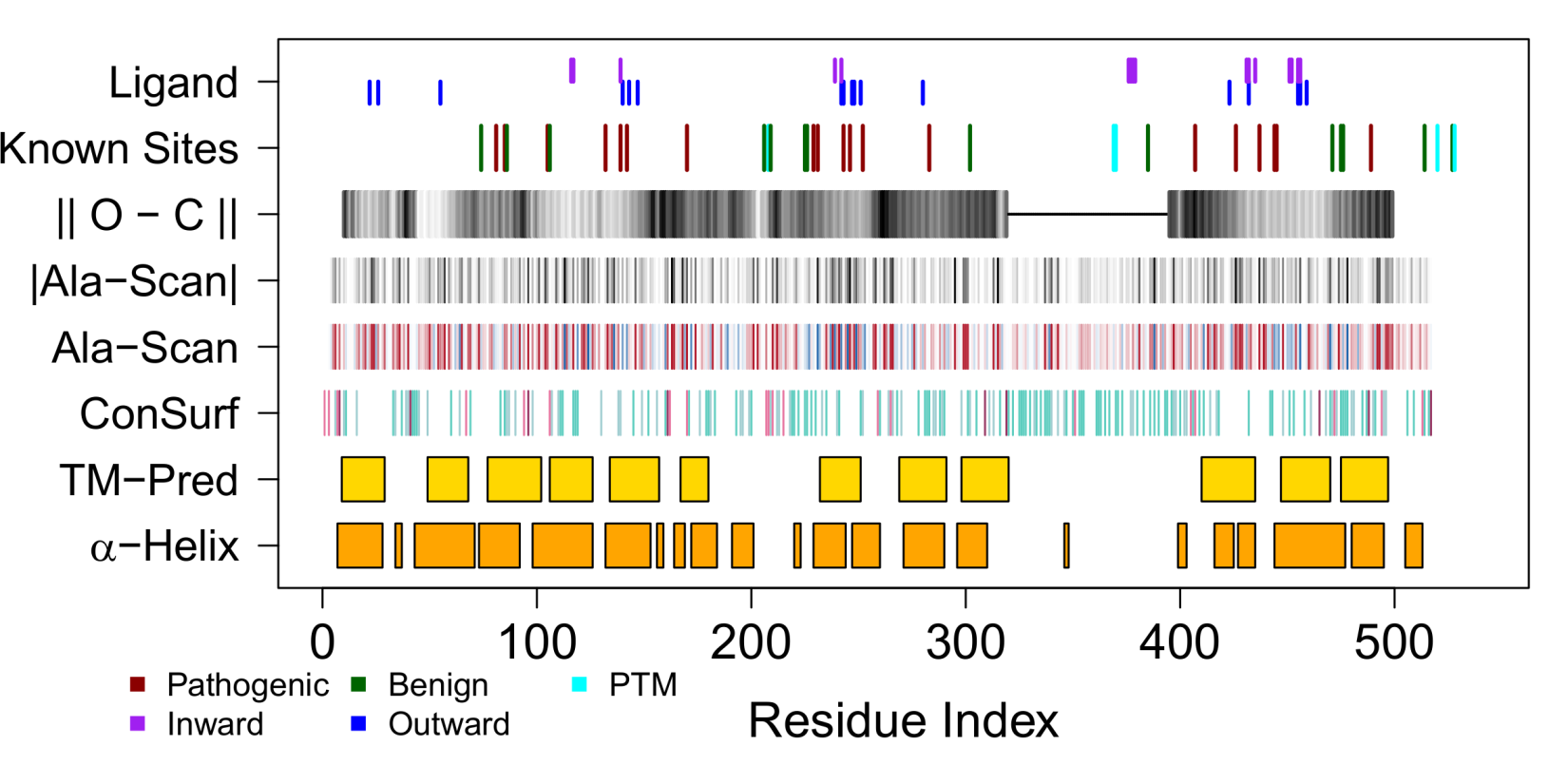


**Figure S2: Multiple types of annotations and predictions shown along the protein sequence reveals relationships between sequential, structural, and dynamical properties.** Each of 8 annotations are shown, each in their own row or “track.” Beginning from the lowest track, we indicate the α-helices within our structural model (outward-facing conformation from the GLUT5 template) and their concordance with consensus TM predictions. Next, the level of per-residue conservation as returned by ConSurf is summarized. The next two tracks show ∆∆G_fold_ from a computational alanine scan; color scale is from blue (-5 kcal/mol) to red (5 kcal/mol). The displacement of C^α^ atoms between the outward-facing and inward-facing is indicated (both using GLUT5 templates), “-“indicating the disordered region. Next, known sites of PTMs or pathogenic and benign variants are indicated. Finally, the top-most track indicates which amino acids are in close contact with α-D-glucose docked to each of the outward-facing and inward-facing models.

**A) B)**


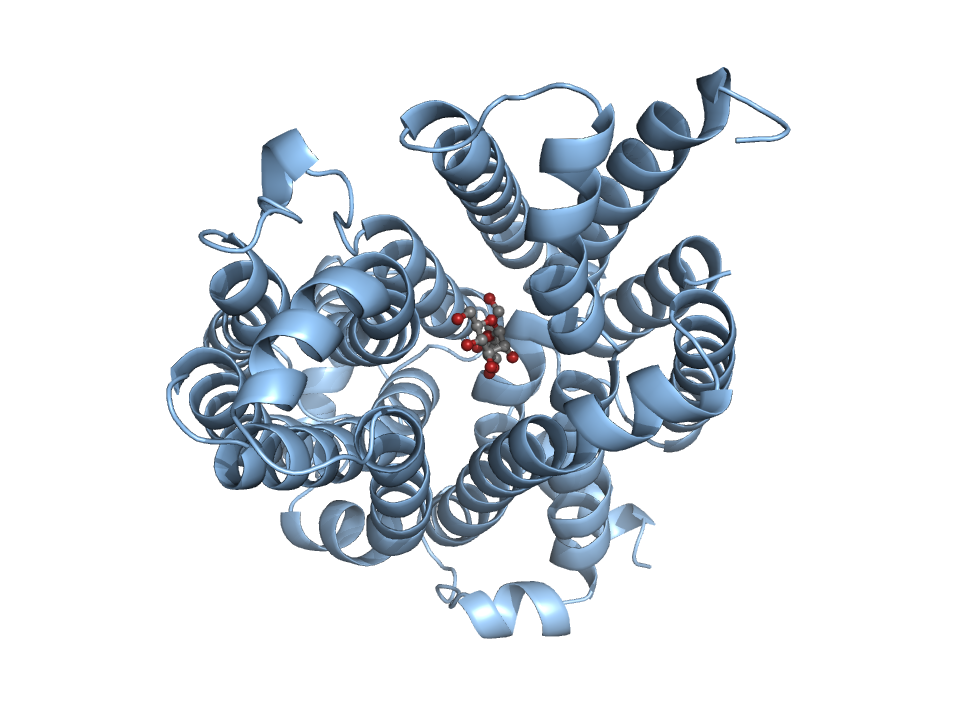

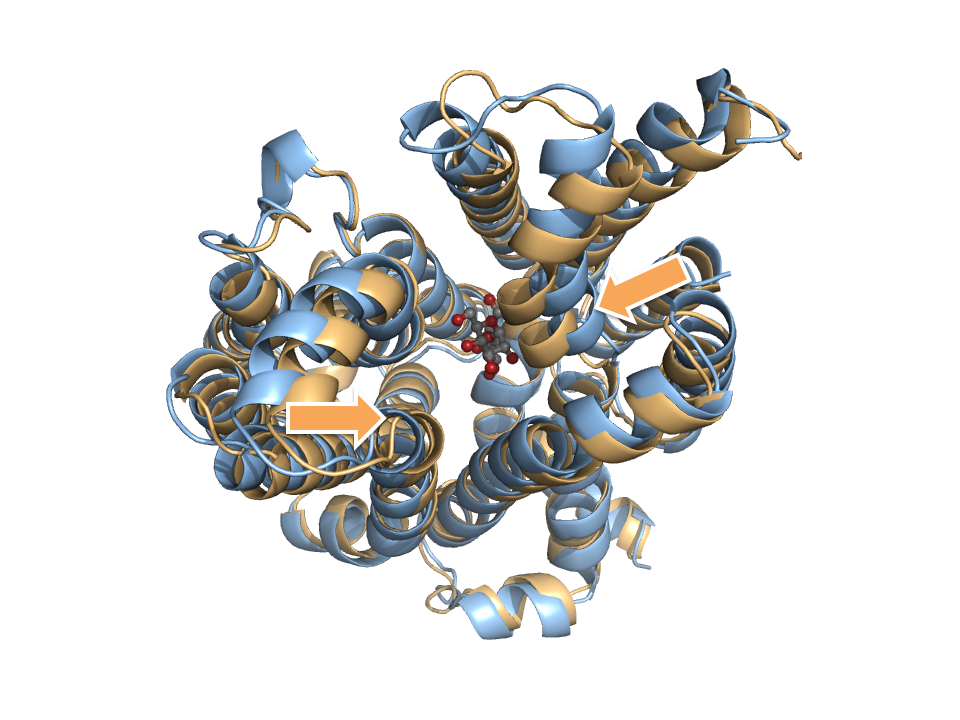


**C) D)**


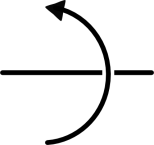

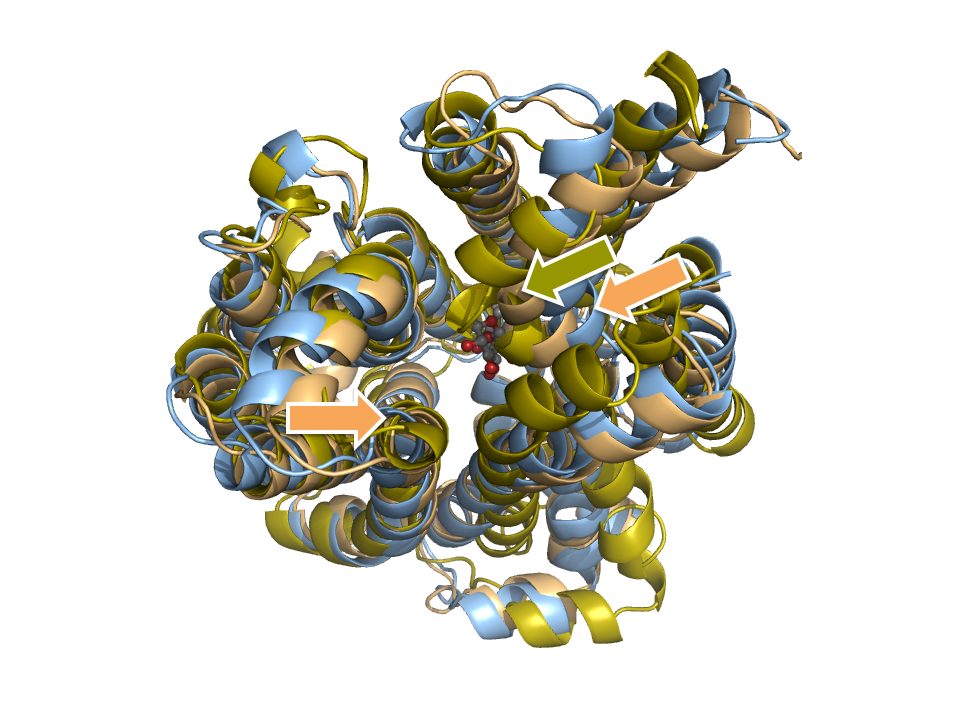

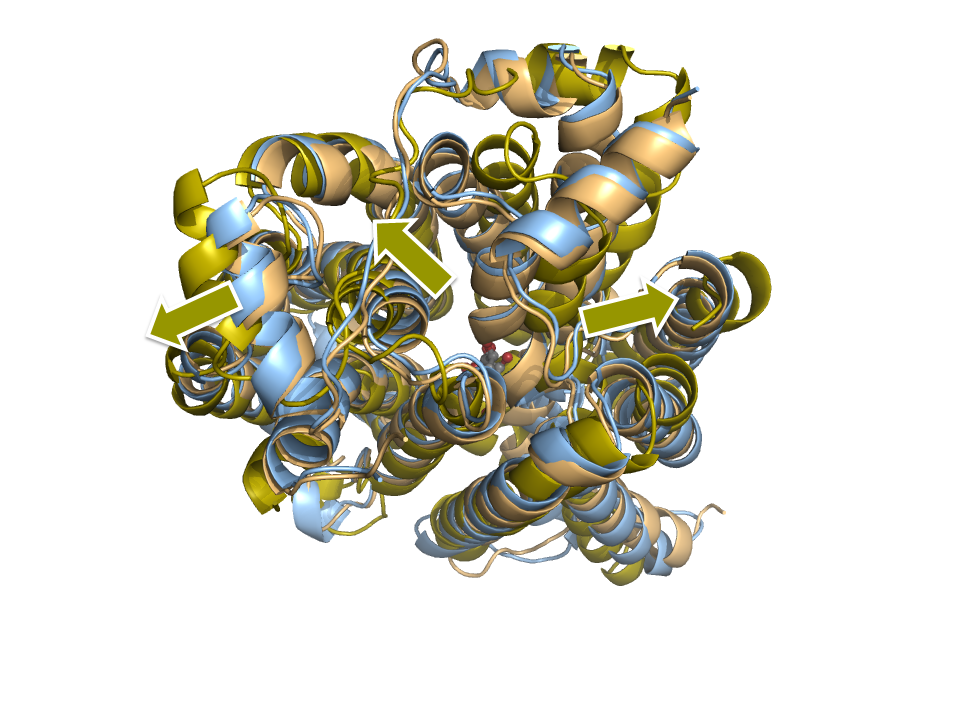


**Figure S3: Experimentally derived GLUT10 models demonstrate conformational diversity.** We show models for the **A)** Outward facing, **B)** outward-occluded and **C, D)** inward-facing conformations in order to indicate the major conformational features of each that have been experimentally observed (indicated by arrows). The outward facing conformation is shown in blue and is present in all panels with the native ligand (maltose) from 4ZWC, for a consistent positional reference. **A)** Viewed from the extracellular side and looking down towards trans-membrane plane, the ligand-binding site is readily accessible. **B)** Upon attaining the outward-occluded state, multiple helices have shifted their position inward. **C)** Finally, in the inward-facing conformation, these helices have shifted even further. **D)** Viewed from the intracellular size, the “opening” conformational change is only evident in the final conformation.


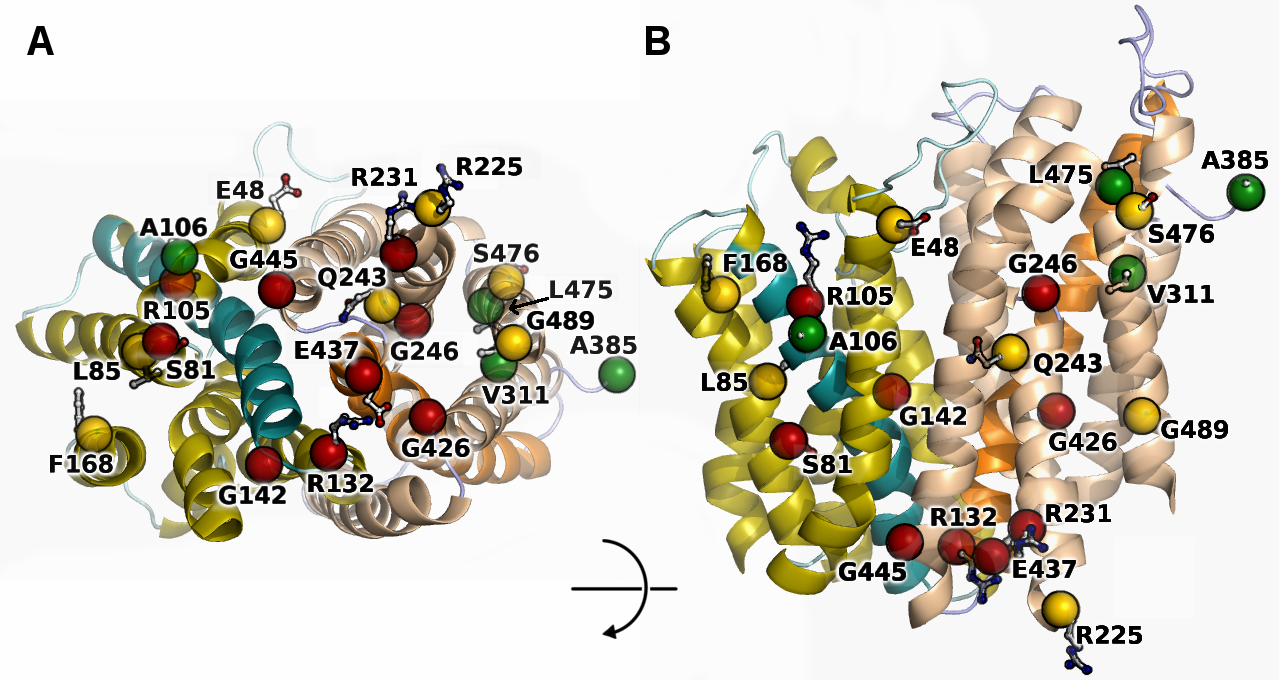


**C**

**
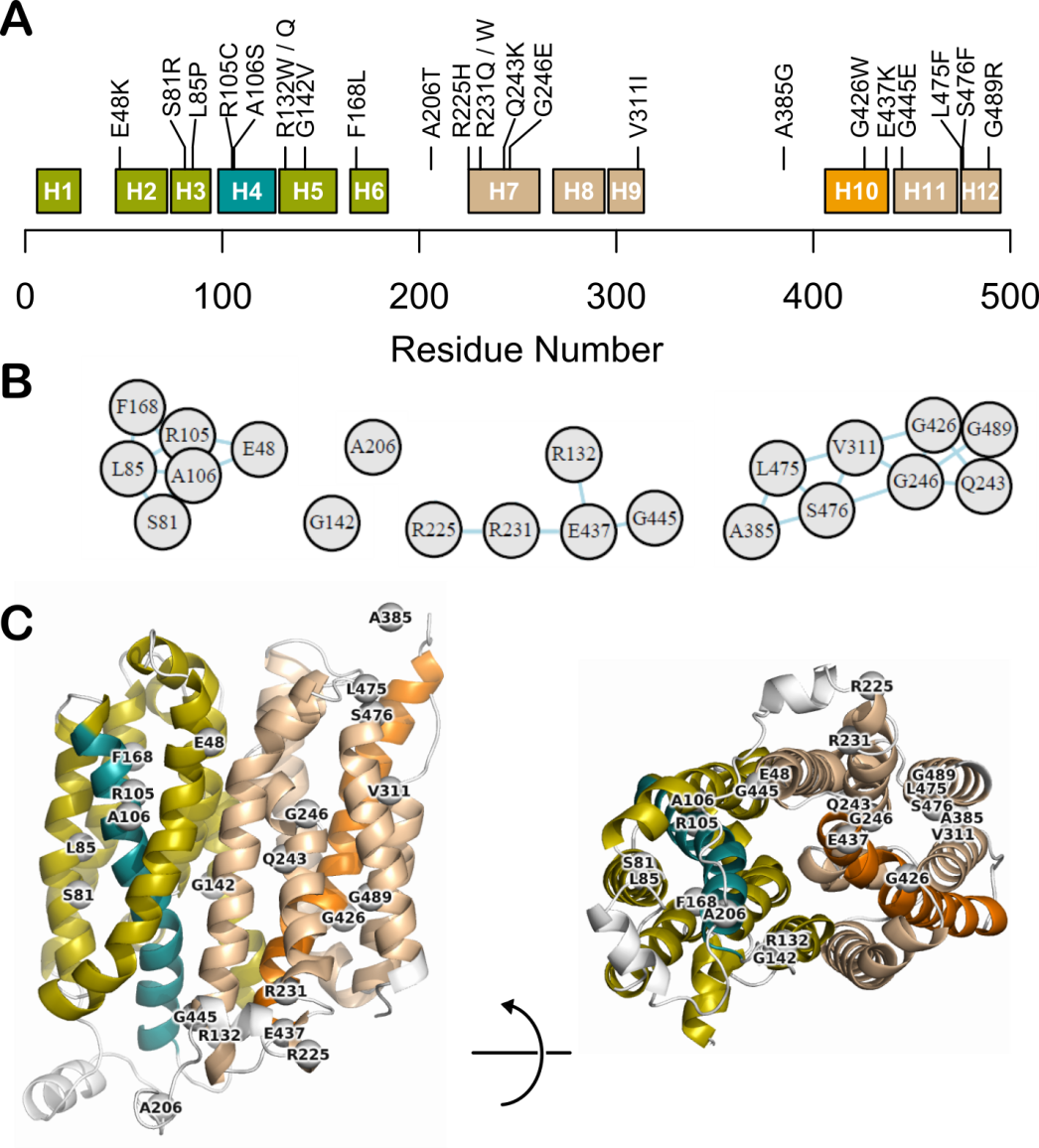
**

**Figure S4: Sites of studied variants throughout GUT10.** We show these known sites highlighted on our outward-facing GLUT10 model. Each mutation site is shown in a ball-and-stick representation with a larger sphere at the C^α^ position. Benign variants are gathered from ClinVar and ExAC (green color). Pathogenic variants are identified from Clinvar, HGMD, or listed in LOVD (red color; gold for VUSs). **C)** While variants are distributed across the linear sequence, they also exhibit 3D relationships. To visualize the spatial proximity of variant sites, we generated a graph where nodes correspond to amino acids and nodes are connected if the amino acids are within 15Å of one another. Three subnetworks are apparent. These are, respectively, a subnetwork within the N-terminal helical bundle, along the intracellular-facing surface, and within the C-terminal helical bundle.

**A**
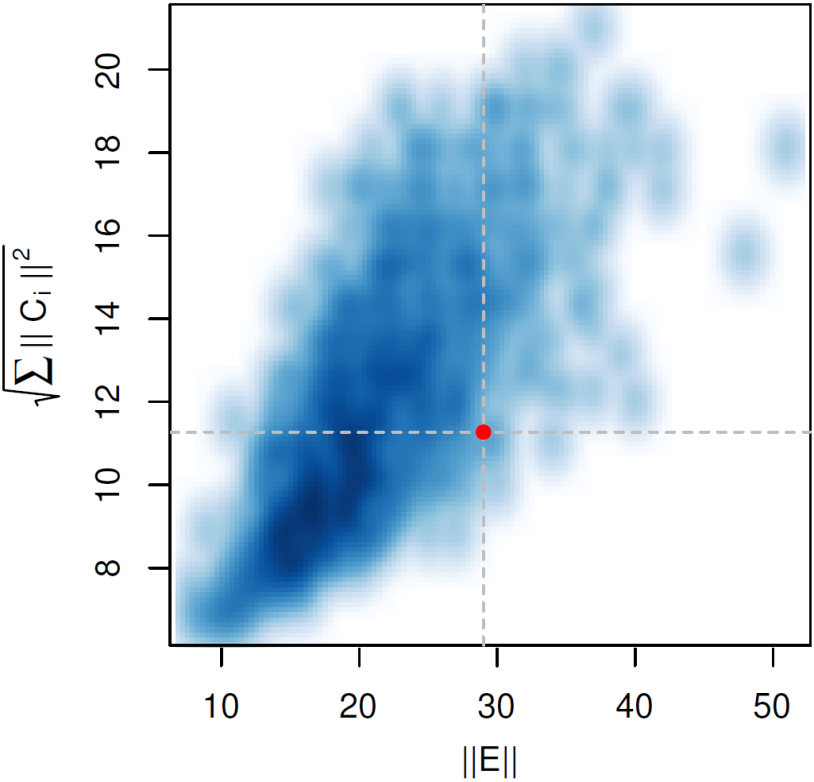


**B
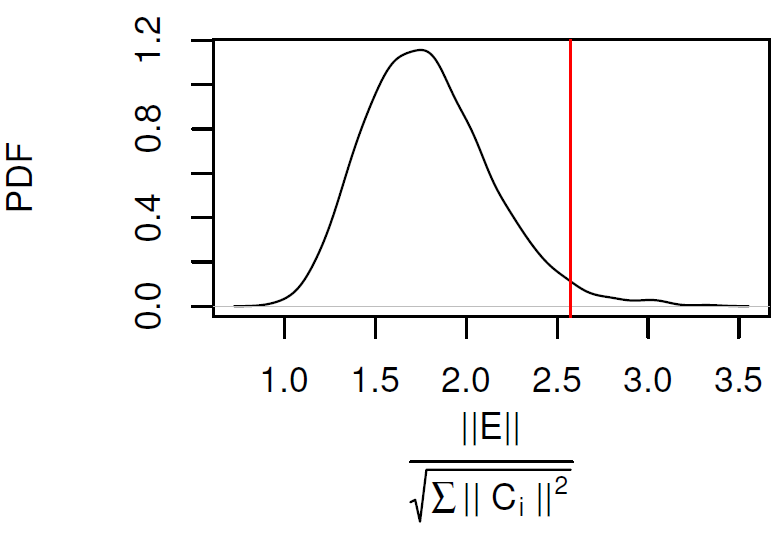
**

**Figure S5: Sites of GLUT10 variants are highly interconnected compared to randomly distributed variants. A)** We randomly permuted the sites of variants and analyzed how clustered they were. The number of residues in the *i*^th^ cluster is designated C_i_ and the number of edges, ||E||. We used the geometric mean of the cluster size distribution as a compactness measure. The distribution of 10,000 permutations is shown as a smoothed density. The observed value for GLUT10 is shown as a red point. Randomly permuted points that occupy the upper-right region (with respect to the grey lines), relative to the observed, are more compact and more dense – they have a greater number of edges and fewer connected clusters. The compactness of observed variants is moderately significant (p = 0.091). Randomly permuted points that occupy the lower-right region, relative to the observed, are more densely connected, but less compact. This pattern indicated a number of densely connected clusters that are well-separated from one another spatially. The observed distribution of variants was significantly dense (p = 0.006), compared to random clusters that were at least as spatially diffuse. **B)** The number of edges, normalized by compactness, was also significant (p = 0.023).


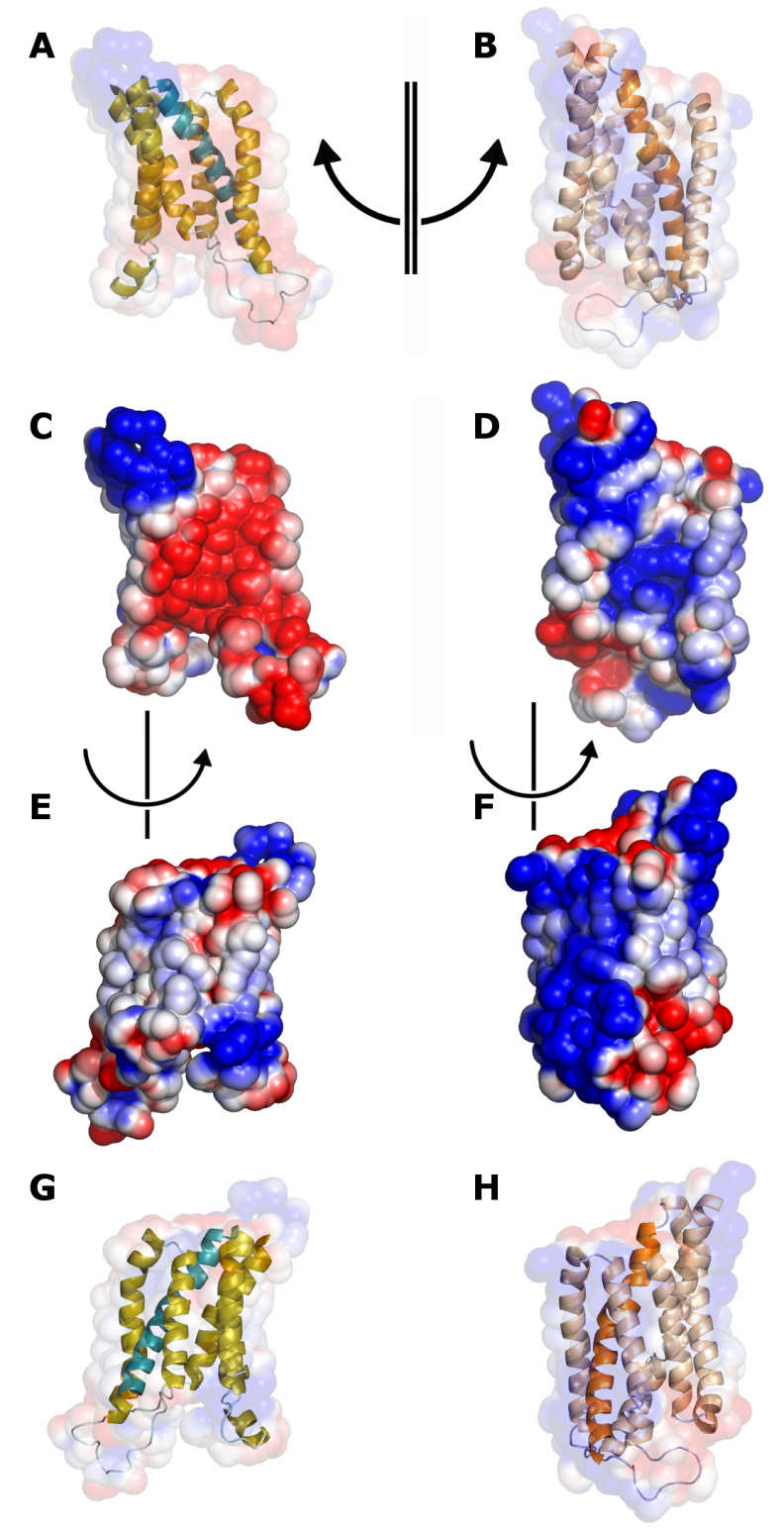


**Figure S6: Subdomain organization coincides with charge separation.** A) The N-terminal helical bundle of our IF model is shown with the surface that faces the C-terminal bundle facing forward. A transparent molecular surface is shown colored by electrostatic potential (red, -2eV; blue +2eV) as calculated by APBS (8). Electrostatic potential surfaces reveal patters between the two subdomains wherein one subdomain presents a mostly positive and the other a mostly negative surface to the other.


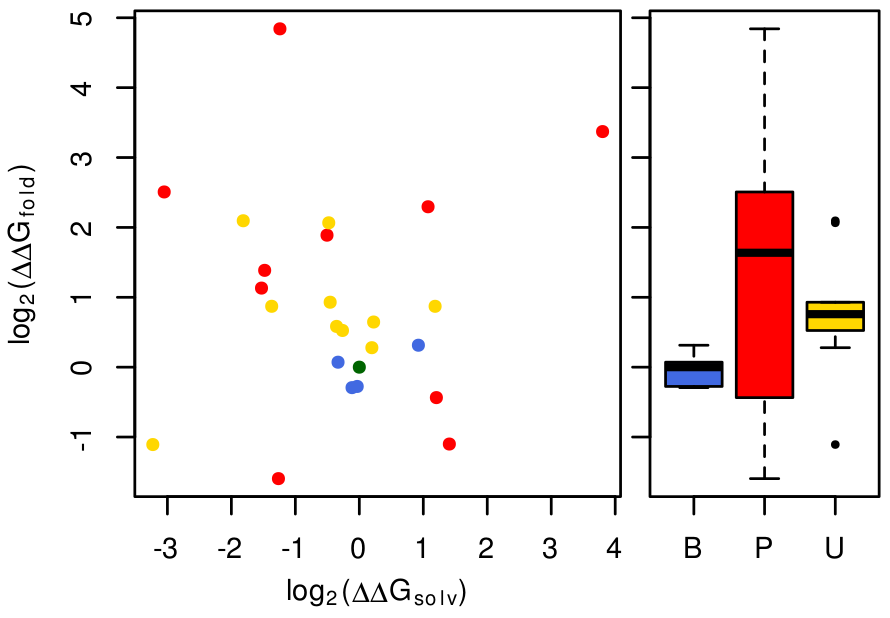


VUS

Pathogenic

Benign

**Figure S7: Mutation of the IF conformation indicates that pathogenic variants tend to be destabilizing.** We indicate variant class by color: blue, benign; red, pathogenic; gold, VUS. Variants of each class are clearly separated from one another by their effect on protein folding energy. Significant differences are observed between each group of variants with known pathogenic variants exhibiting greater ΔΔG_fold_ than benign variants (p = 0.012). Many VUSs also destabilize the protein structure (p = 0.024), but to a lesser degree compared to established pathogenic variants. The relationship between destabilization (ΔΔG_fold_) and changes in solvation energy (ΔΔG_solv_), where some destabilizing variants increase solvation energy and others decrease it, emphasizes that there are multiple ways to alter protein structure and single measures may be inadequate to resolve mechanisms.

**
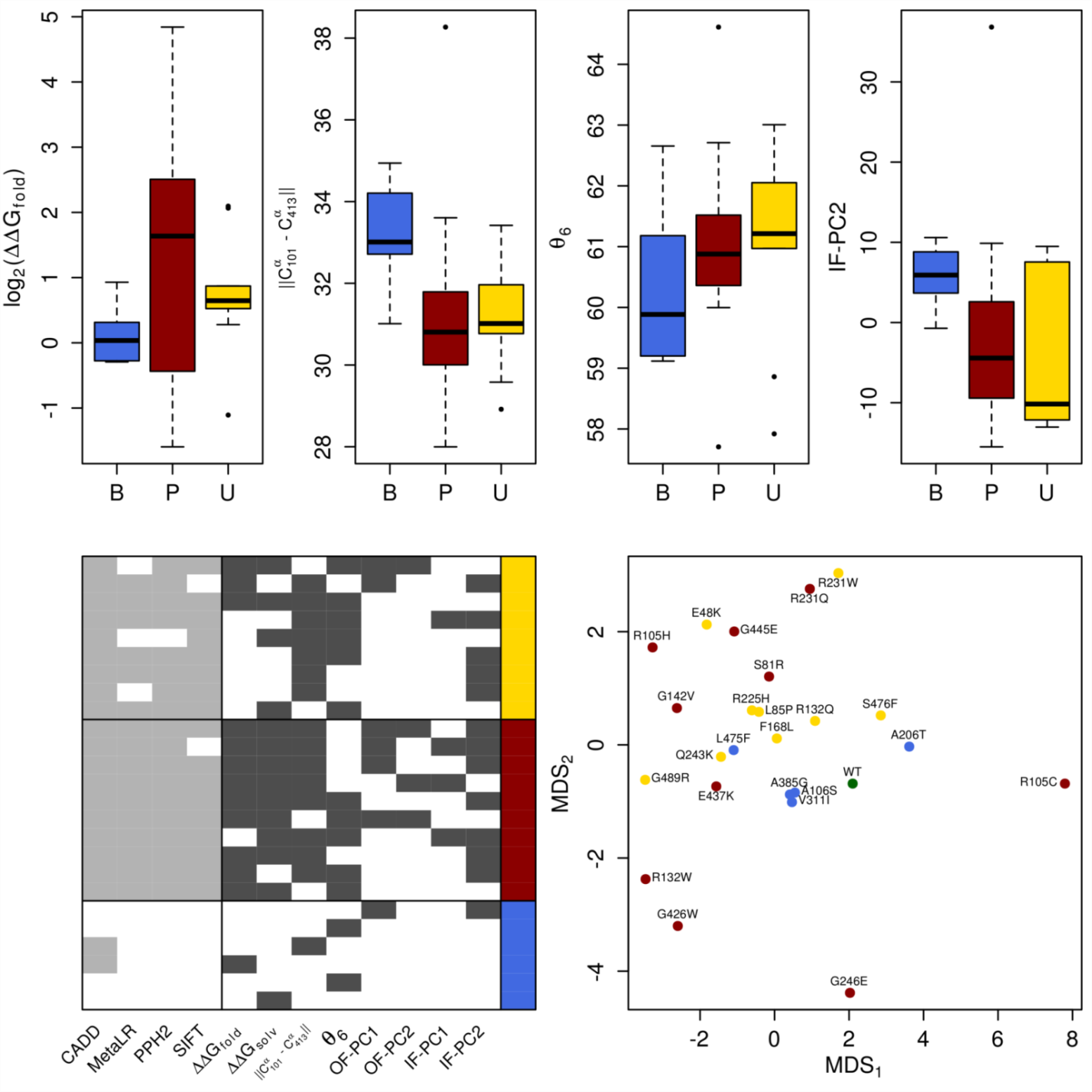
**

**Figure S8: Examples of potentially discriminatory structure-based metrics.** Distance and angle monitors were used to identify thresholds that distinguish WT-like dynamic patters. Variant classes are indicated along the abscissa: benign, B; pathogenic, P; uncertain, U. These metrics and others are used to compare among variants in Figure 5.


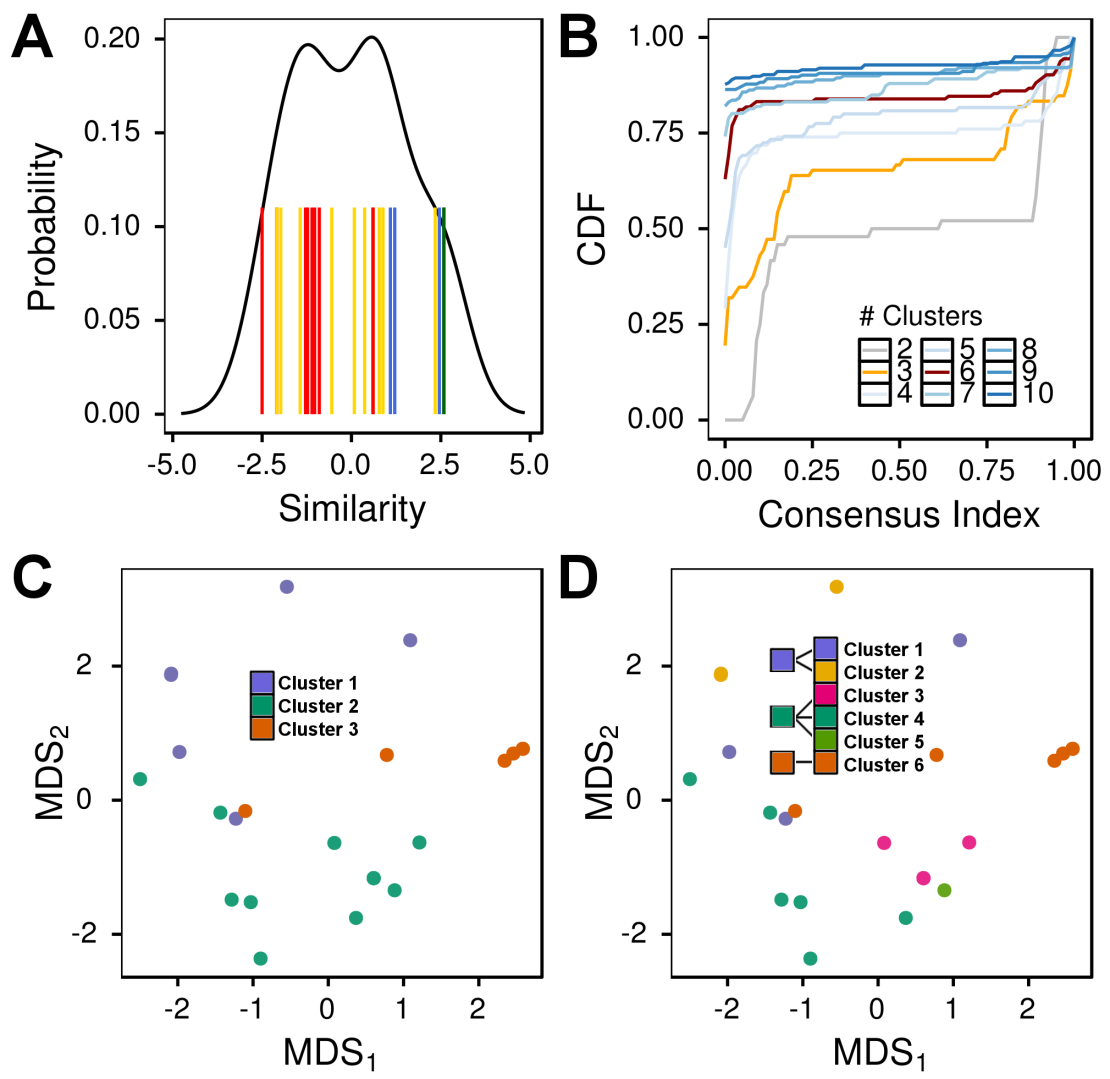


MDS_1_

**Figure S9: Structure-based metrics discriminate pathogenic from benign variants and provide mechanistic hypothesis of altered function. A)** Variants are ordered by their similarity as summarized by Multidimensional Scaling (see Methods and Figure 5). Benign variants are on one side and separated from pathogenic variants. **B)** We used consensus clustering to group variants by similarity across sequence- and structure-based metrics. We measured the consensus Cumulative Distribution Function (CDF; measure of how frequently the same variants appear in the same clusters) for *k* = 2-10 clusters, and 100 iterations for each value of *k*, Significant gains in cluster consistency are observed when grouping the data into either three or six clusters. **C,D)** The same MDS data in Figure 5 is visualized and colored by cluster identity from consensus clustering using three clusters or six clusters.

# Supplemental References

1. Deng D, Xu C, Sun P, Wu J, Yan C, Hu M, et al. Crystal structure of the human glucose transporter GLUT1. *Nature* (2014) 510(7503):121-5. Epub 2014/05/23. doi: 10.1038/nature13306. PubMed PMID: 24847886.

2. Deng D, Sun P, Yan C, Ke M, Jiang X, Xiong L, et al. Molecular basis of ligand recognition and transport by glucose transporters. *Nature* (2015) 526(7573):391-6. doi: 10.1038/nature14655. PubMed PMID: 26176916.

3. Nomura N, Verdon G, Kang HJ, Shimamura T, Nomura Y, Sonoda Y, et al. Structure and mechanism of the mammalian fructose transporter GLUT5. *Nature* (2015) 526(7573):397-401. doi: 10.1038/nature14909. PubMed PMID: 26416735; PubMed Central PMCID: PMCPMC4618315.

4. Quistgaard EM, Low C, Moberg P, Tresaugues L, Nordlund P. Structural basis for substrate transport in the GLUT-homology family of monosaccharide transporters. *Nat Struct Mol Biol* (2013) 20(6):766-8. Epub 2013/04/30. doi: 10.1038/nsmb.2569. PubMed PMID: 23624861.

5. Sun L, Zeng X, Yan C, Sun X, Gong X, Rao Y, et al. Crystal structure of a bacterial homologue of glucose transporters GLUT1-4. *Nature* (2012) 490(7420):361-6. Epub 2012/10/19. doi: 10.1038/nature11524. PubMed PMID: 23075985.

6. Dang S, Sun L, Huang Y, Lu F, Liu Y, Gong H, et al. Structure of a fucose transporter in an outward-open conformation. *Nature* (2010) 467(7316):734-8. Epub 2010/09/30. doi: 10.1038/nature09406. PubMed PMID: 20877283.

7. Willard L, Ranjan A, Zhang H, Monzavi H, Boyko RF, Sykes BD, et al. VADAR: a web server for quantitative evaluation of protein structure quality. *Nucleic Acids Res* (2003) 31(13):3316-9. PubMed PMID: 12824316; PubMed Central PMCID: PMCPMC168972.

8. Baker NA, Sept D, Joseph S, Holst MJ, McCammon JA. Electrostatics of nanosystems: application to microtubules and the ribosome. *Proc Natl Acad Sci U S A* (2001) 98(18):10037-41. Epub 2001/08/23. doi: 10.1073/pnas.181342398. PubMed PMID: 11517324; PubMed Central PMCID: PMC56910.
